# Evolutionary trajectories of β-lactam resistance in *Enterococcus faecalis* strains

**DOI:** 10.1128/mbio.02897-24

**Published:** 2024-11-14

**Authors:** Paul Ugalde Silva, Charlene Desbonnet, Louis B. Rice, Mónica García-Solache

**Affiliations:** 1 Department of Medicine, Rhode Island Hospital, Warren Alpert Medical School of Brown University, Providence, Rhode Island, USA; Universite de Geneve, Geneva, Switzerland

**Keywords:** *Enterococcus*, beta-lactams, drug resistance evolution, antibiotic dependence, c-di-AMP, long-term evolution experiment

## Abstract

Resistance to ampicillin and imipenem in *Enterococcus faecalis* is infrequent. However, the evolution of resistance can occur through prolonged antibiotic exposure during the treatment of chronic infections. In this study, we conducted a long-term evolution experiment using four genetically diverse strains of *E. faecalis* with varying susceptibilities to ampicillin and imipenem. Each strain was subjected to increasing concentrations of either ampicillin or imipenem over 200 days, with three independent replicates for each strain. Selective pressure from imipenem led to the rapid selection of highly resistant lineages across all genetic backgrounds, compared to ampicillin. In addition to high resistance, we describe, for the first time, the evolution of a β-lactam-dependent phenotype observed in lineages from all backgrounds. Whole-genome sequencing and bioinformatic analysis revealed mutations in three main functional classes: genes involved in cell wall synthesis and degradation, genes in the walK/R two-component system, and genes in the c-di-AMP pathway. Our analysis identified new mutations in genes known to be involved in resistance as well as novel genes potentially associated with resistance. Furthermore, the newly described β-lactam-dependent phenotype was correlated with the inactivation of c-di-AMP degradation, resulting in high levels of this second messenger. Together, these data highlight the diverse genetic mechanisms underlying resistance to ampicillin and imipenem in *E. faecalis*. The emergence of high resistance levels and β-lactam dependency underscores the importance of understanding evolutionary dynamics in the development of antibiotic resistance.

**IMPORTANCE:**

*Enterococcus faecalis* is a major human pathogen, and treatment is frequently compromised by poor response to first-line antibiotics such as ampicillin. Understanding the factors that play a role in susceptibility/resistance to these drugs will help guide the development of much-needed treatments.

## INTRODUCTION

*Enterococcus faecalis* is one of the leading causes of healthcare-associated infections (1, 2). The widespread use of antibiotics in various human activities has led to the selection of strains equipped with mechanisms to resist antibiotic treatments to which they were previously susceptible. The increasing emergence of antibiotic resistance in this commonly occurring pathogen (3) represents a clinical challenge and is associated with worse clinical outcomes when compared with susceptible strains (4, 5). Mortality rates of enterococcal bacteremia range from 20% to 50%, and patient comorbidities and antimicrobial resistance are the major risk factors for early mortality (6).

*E. faecalis* expresses inherent resistance to many β-lactam treatments, although the extent of resistance varies depending on the specific class of β-lactam. Amino-penicillins, such as ampicillin, are highly effective, while carbapenems, like imipenem, exhibit lower efficacy, and cephalosporins are the least effective (7). In clinical practice, ampicillin is considered the treatment of choice for infections caused by susceptible *E. faecalis* strains (8); however, we and others have reported clinical strains showing ampicillin resistance (9–12). Imipenem, although not specifically indicated for enterococcal infections, demonstrates *in vitro* activity against many strains and is sometimes used (13–15).

Development of resistance to ampicillin and imipenem during typical courses of antimicrobial therapy is rare in *E. faecalis* (16, 17). However, the evolution of resistance to β-lactams may be associated with prolonged antibiotic exposure during the treatment of chronic infections. Previously, we reported a clinical isolate of *E. faecalis* obtained from a patient who had undergone extended amoxicillin therapy, which was resistant to ampicillin and penicillin. The isolate carried mutations in one of the main determinants for β-lactam resistance, the penicillin-binding protein 4 gene (*pbp4*). These changes led to a reduction in Pbp4’s affinity for β-lactams and triggered its overproduction (9). While mutations in *pbp4* conferred an advantage during selection pressure resulting in the emergence of resistance, they were associated with reduced bacterial growth rate and cell wall stability.

Resistance and the associated fitness costs may arise from diverse evolutionary trajectories, influenced by the cooperative effects of acquired mutations and the underlying genetic backgrounds (18–20). Consequently, predicting the specific trajectories leading to the evolution of resistance poses a significant challenge (21, 22). Aside from *pbp4* mutations, little is known about additional mechanisms of ampicillin resistance in *E. faecalis*. Our goal in the present study was to identify convergent and novel pathways to β-lactam resistance in *E. faecalis* strains with diverse genetic backgrounds.

To understand the evolutionary trajectories leading to β-lactam resistance in *E. faecalis*, we conducted a long-term evolution experiment (LTEE) using four distinct strains originating from different sources subjected to escalating concentrations of either ampicillin or imipenem over a period of 200 days. Through this experiment, we validated previously described genes leading to resistance to β-lactams, identified novel pathways, and determined that although there were convergent pathways, each strain follows a different evolutionary trajectory. Our findings underscore the significance of genetic background in predicting specific resistance patterns and highlight the genetic changes associated with fitness costs imposed during the evolution of resistance.

## RESULTS

### Selection of β-lactam-resistant *E. faecalis* in a LTEE

We set up an LTEE using four different *E. faecalis* strains from different sources and genetic backgrounds (Table 1). We obtained a total of 432 samples from our initial passages at the end of the experiment. Additionally, we inoculated a separate plate to initiate a parallel LTEE (replica cultures) on day 68 to investigate the reproducibility of the path to resistance in each strain. From the replicate cultures, we obtained a total of 252 samples. The total number of antibiotic-selected lineages obtained in our experiment was 48 (Table S1).

**TABLE 1:** Strains used in the LTEE

Strain	Source	Amp MIC	Imi MIC	Reference(s)
LS4828	Human—prosthetic knee joint	16	16	(9)
ATCC 29212	Human—urine	2	2	(23)
JH2-2	Human—stools	1.5	1.5	(24, 25)
D32	Porcine—stools	2	2	(26)

### Evolution of β-lactam resistance during the LTEE

Periodic ampicillin and imipenem susceptibility testing during the experiment revealed a progressive loss of susceptibility to ampicillin and imipenem under both antibiotics’ selection.

**Fig 1:**
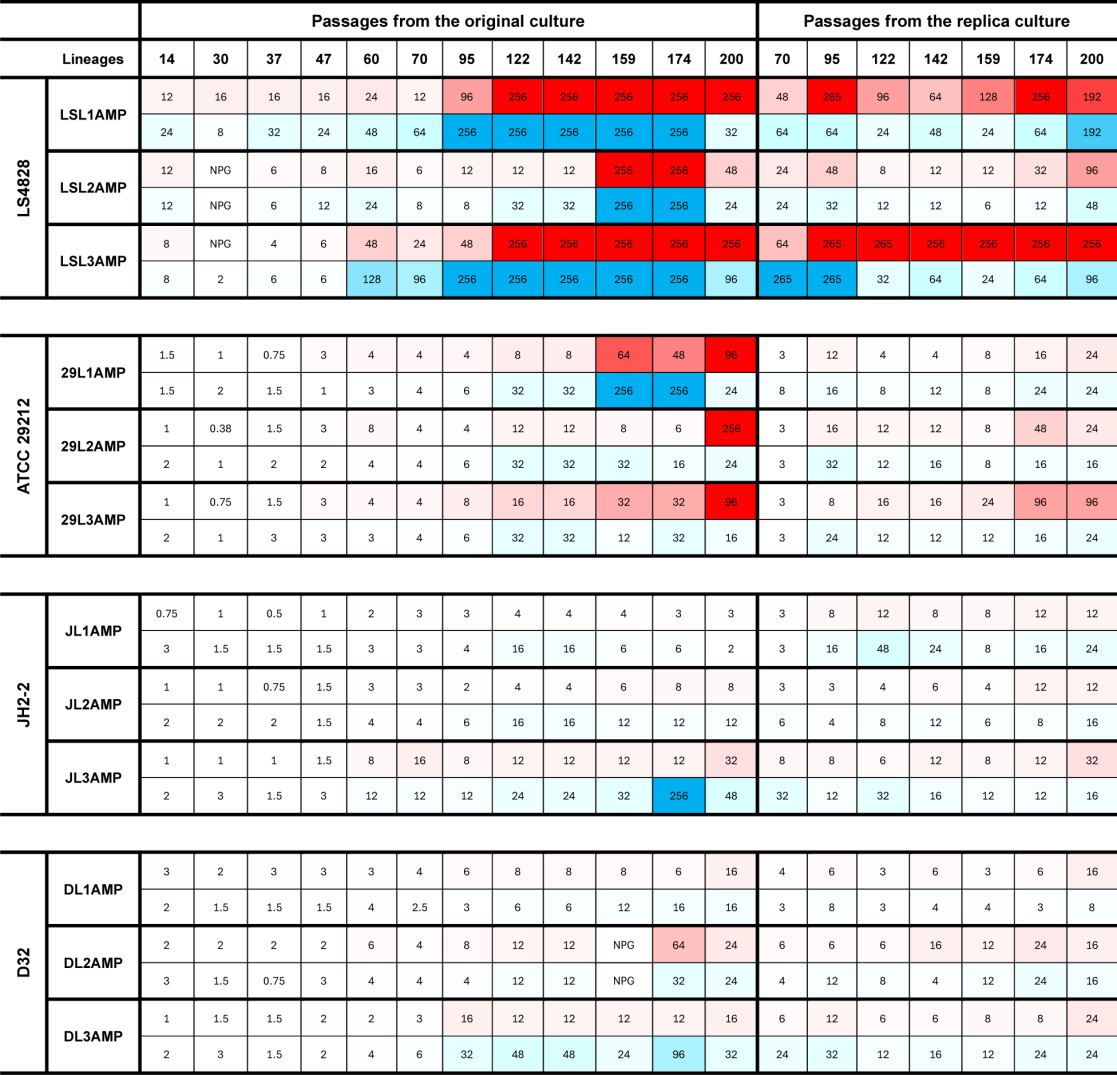
Ampicillin and imipenem MICs from lineages selected with ampicillin. Heat map of the minimal inhibitory concentration (MIC) of ampicillin (red) and imipenem (blue) obtained from the lineages evolved under ampicillin exposure. MICs were determined between 18 and 24 h of incubation and read by eye. Squares with a diagonal solid line reflect passages at which the MIC could not be obtained between 18 and 24 h. Strain designation: LS lineages are derived from LS4828; 29 lineages are derived from ATCC 29212; J lineages are derived from JH2-2; D lineages are derived from D32. Lineage designation: first initial describes strain name, second initial and number describe lineage number; amp or imi informs antibiotic selection. Ampicillin MICs are highlighted in red; imipenem MICs are highlighted in blue. NPG, no perceptible growth.

Ampicillin exposure selected for ampicillin resistance in the three ATCC 29212 and D32 lineages and in two of three JH2-2 lineages in both original and replica plates; additionally, all ampicillin-selected lineages evolved imipenem resistance (Fig. 1). Resistance to ampicillin and imipenem (MIC ≥ 8 µg/mL) in the lineages from the susceptible strains ATCC 29212 (23), JH2-2 (24), and D32 (26) appeared later during the experiment (around passage 122).

**Fig 2:**
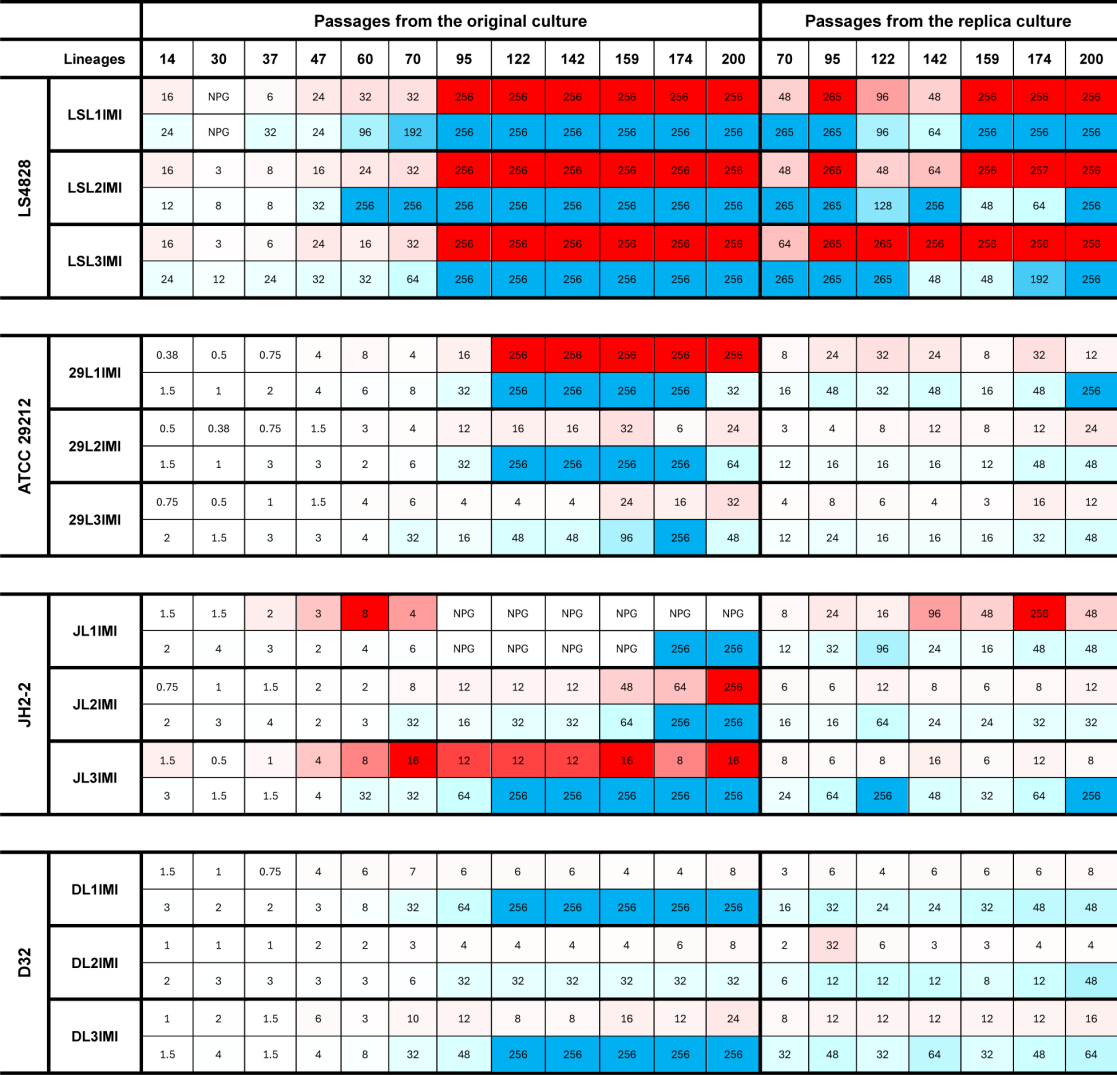
Ampicillin and imipenem MICs from lineages selected with imipenem. Heat map of the minimal inhibitory concentration (MIC) of ampicillin (red) and imipenem (blue) obtained from the lineages evolved under imipenem exposure. MICs were determined between 18 and 24 h of incubation and read by eye. Squares with a diagonal solid line reflect passages in which the MIC could not be obtained between 18 and 24 h. Ampicillin MICs are highlighted in red; imipenem MICs are highlighted in blue; NPG, no perceptible growth.

Lineages under imipenem selection acquired higher MICs to both imipenem and ampicillin earlier in the experiment compared to ampicillin selection (Fig. 2). In the susceptible backgrounds, most displayed higher MICs to imipenem than to ampicillin in both original and replica cultures. Cross-resistance to ampicillin evolved in most lineages (Fig. 2); resistance to ampicillin in D32-derived lineages was slow to evolve compared to the other backgrounds. Interestingly, the breakpoint (≥8 µg/mL) was reached in most cases between passages 95 and 122.

LS4828, which was resistant to ampicillin and imipenem at the start, evolved increased resistance to both antibiotics, reaching MICs ≥ 256 µg/mL.

Our data indicate that continuous imipenem exposure selects for the evolution of high-level ampicillin and imipenem resistance at a higher frequency than ampicillin exposure. Imipenem-selected lineages show higher variability in the MIC distribution and timing of β-lactam resistance acquisition. Imipenem selection tends to favor the rapid evolution of highly resistant lineages, compared to ampicillin selective pressure, across all tested genetic backgrounds. The evolution of ampicillin resistance seems to carry stronger fitness costs than imipenem exposure since it is slower to evolve, and the MICs tend to be lower than lineages evolved under imipenem.

In the lineages passaged under no antibiotic pressure (Table S2), two lineages from the resistant strain LS4828 lost resistance to ampicillin and imipenem (MICs between 0.5 and 3 µg/mL). The ampicillin and imipenem MICs of the lineages from the susceptible backgrounds did not change.

Our findings demonstrate a remarkable ability of *E. faecalis* to adapt to elevated concentrations of β-lactams while also losing resistance if the selection pressure is removed, suggesting that β-lactam resistance carries fitness costs, as demonstrated by a lower growth rate of LS4828 and all resistant lineages compared to susceptible strains (data not shown).

### Chronic exposure to ampicillin and imipenem selected the evolution of β-lactam dependence

**Fig 3:**
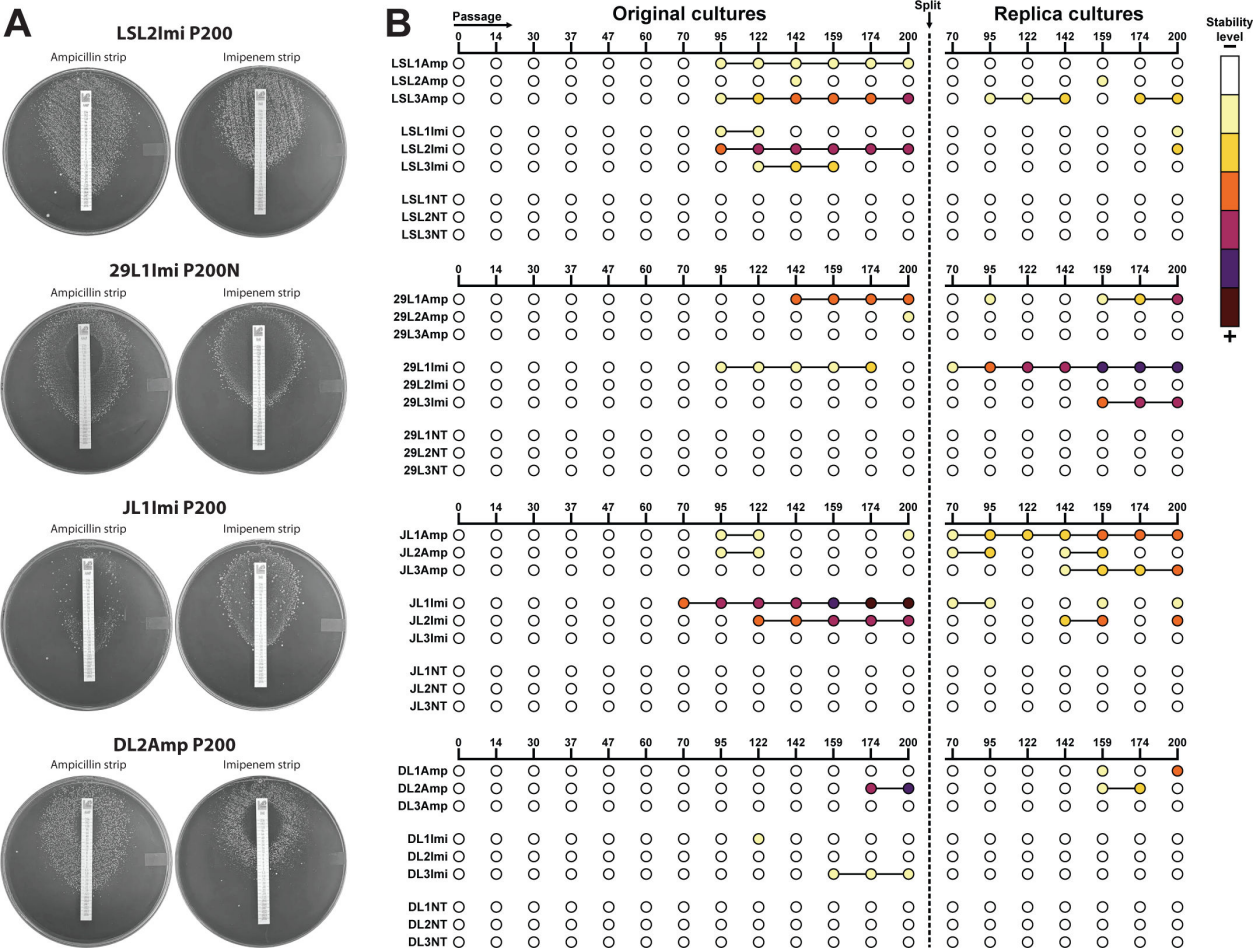
β-Lactam-dependent lineages. (**A**) MIC strip tests of strongly dependent passage 200 lineages. LSL2Imi P200 (LS4828), 29L1Imi P200R (ATCC29212, replica culture), JL1Imi P200 (JH2-2), and DL2Amp P200 (D32). The plates show dense areas of growth near the test strip and an area devoid of colonies in the lower antibiotic concentrations and away from the strip. (**B**) Dot chart highlighting β-lactam dependence during the experiment. Light to dark color shows the progression of the phenotype and how stable it was as determined by (i) dependence is present at 24 h but lost at 72 h and all colonies are homogenous; (ii) dependence is present at 24 h but lost at 72 h, two types of colonies; (iii) dependence is present at 24 and 72 h, two types of colonies.

During the LTEE, in addition to the evolution of β-lactam resistance, we identified an unexpected β-lactam-dependent phenotype in 30 resistant lineages from all genetic backgrounds. This phenotype was observed as a dense zone of bacterial growth (growth halo) near the highest antibiotic concentrations of the MIC test strip, with little to no colonies observed in the proximity of the lower antibiotic concentrations or away from the strip (Fig. 3A). Dependence on the antibiotic to grow was observed as early as passage 70 and after the acquisition of resistance. In seven of the lineages, it was later lost (Fig. 3B). In general, the dependent phenotype was more stable in later passages, with less colonies appearing on the periphery of the plate away from the disc (data not shown).

To test the stability of the dependent phenotype, we performed growth curves with CFU counts for four antibiotic-dependent lineages at passage 200; cultures were supplemented with either ampicillin or imipenem or grown without antibiotics (Fig. S2A). The dependent lineages grew faster in the presence of either ampicillin or imipenem, although ampicillin-grown cells presented a slower growth rate and reached lower CFUs (Fig. S2A). The absence of antibiotics negatively impacted the growth of the four lineages. The lineages grown in the presence of β-lactams maintained a dependent phenotype, forming growth halos around the discs (ampicillin, imipenem, and penicillin). The four lineages lost antibiotic dependence when they were grown in the absence of β-lactams. All lineages maintained β-lactam resistance (Fig. S2B).

Since selection with ampicillin or imipenem resulted in cross-resistance to the other antibiotic and penicillin, we tested 13 different β-lactams for cross-resistance and dependence against four dependent lineages by disc diffusion assay (Table S3). The four lineages formed robust growth halos around penicillin, ampicillin, and imipenem discs, and around two other penicillins: ticarcillin and piperacillin. Growth around cephalosporins was more variable, less robust, and slower. None of the lineages grew around oxacillin, cefotetan, or vancomycin discs (Table S3).

### Genome evolution under ampicillin and imipenem selection

We sequenced a total of 135 genomes: 46 derived from LS4828, 21 from D32, 24 from ATCC 29212, and 44 from JH2-2 from our LTEE, but we discarded four that were found to be a cross-contamination (Table S1). We also sequenced our laboratory stocks for the parental strains for accurate variant calling.

**Fig 4:**
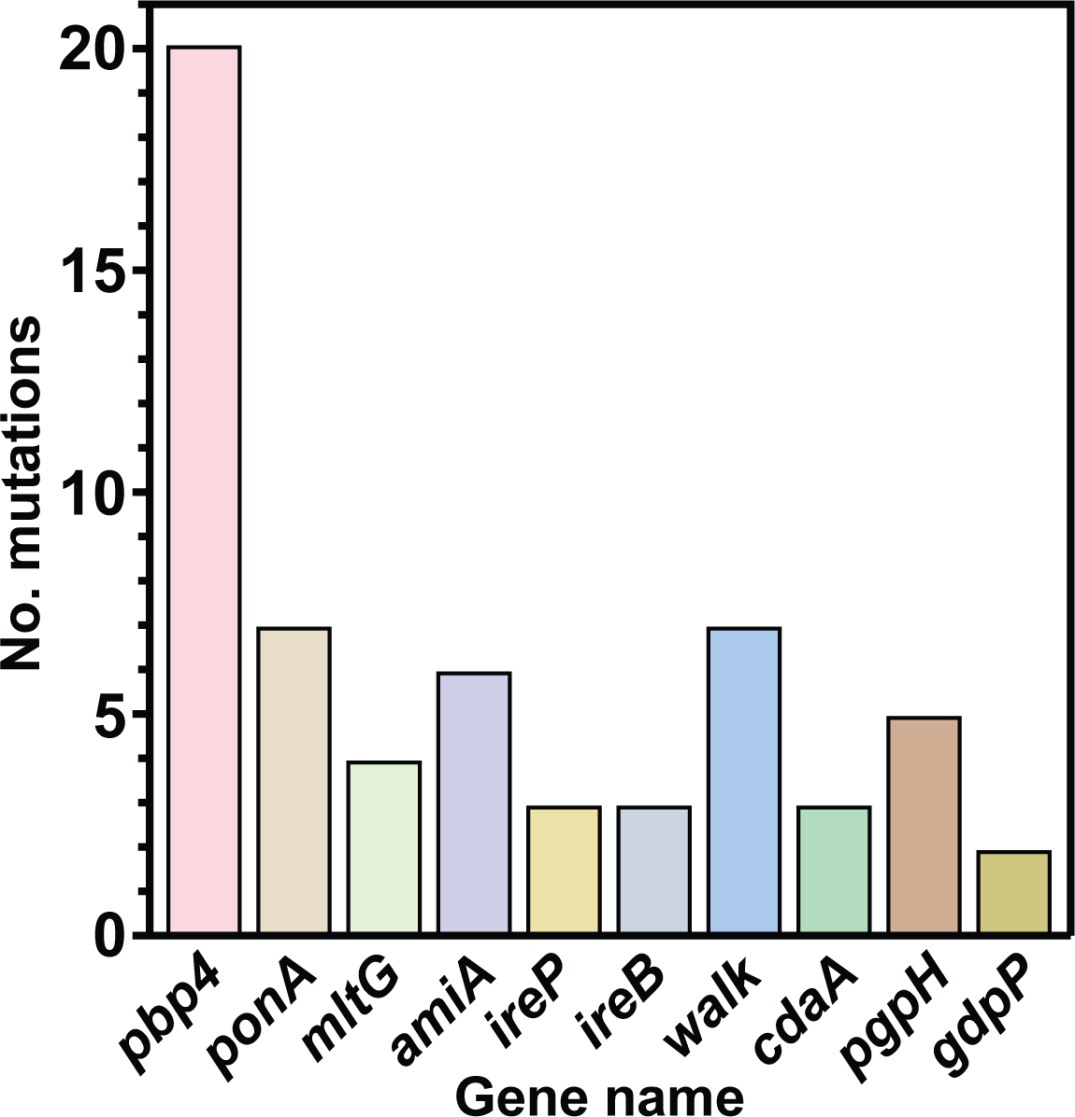
Mutations in cell wall-regulating genes. The graph shows the number of mutations identified in genes involved in cell regulation that were present in more than one lineage.

Lineages derived from the resistant strain LS4828 accumulated less mutations compared with the lineages derived from the susceptible strains (Fig. S3). Deletions > 50 bp were rare in most genomes, except for DL2Amp, which lost a 12,895-bp plasmid, losing 16 genes. After the culture split, DL2AmpR started to accumulate mutations between passages 95 and 122, coincidental with a 10-bp deletion in the DNA mismatch repair protein MutS gene. Mutations in *mutS* and *mutL* have been correlated with an increase in the spontaneous mutation rate in *E. faecalis* (27). Although each susceptible strain followed a different evolutionary trajectory to acquire β-lactam resistance, we identified a core of genes mutated in more than one lineage (Fig. 4), suggesting their importance in either resistance or fitness as described below.

Our LTEE allowed us to describe, for the first time, the mutational steps involved in the evolution of very high levels of resistance to ampicillin and imipenem (MICs ≥ 50 µg/mL) in *E. faecalis*.

### Mutations in genes related to cell wall metabolism

The main target for β-lactams is the cell wall-building penicillin-binding proteins (Pbps). During the experimental evolution of β-lactam resistance, we identified different mutations affecting the genes for these enzymes and other genes involved in cell wall homeostasis in all the sequenced lineages (Fig. 4; Table S4).

In *E. faecalis,* Pbp4 has been described as the main determinant for β-lactam resistance, and as such, we identified mutations in the *pbp4* gene in resistant lineages derived from the three susceptible backgrounds; we did not identify new *pbp4* mutations in lineages derived from LS4828. We found two different kinds of mutations: those affecting the promoter region and those affecting the coding region. We identified three mutation sites in the promoter: the deletion of an A at positions –57/–51 and –88/–81, and a nucleotide substitution at position −46 (relative to the start codon of the gene) (Fig. S4A).

In the *pbp4* coding region, we identified seven different mutations located in the transpeptidase domain. Three different mutation sites were identified independently in more than one lineage (Fig. S4B). Only one of the changes identified in this work coincided with previously described mutations (28); however, we previously reported a mutation in position 617 of the protein in LS4828, which was correlated with decreased penicillin binding (9), and in this current work, we identified a different change in the same position (Table S4). In addition to *pbp4* mutations, we identified five lineages with mutations in the class A Pbp *ponA* gene. All mutations were within the putative transpeptidase domain (Fig. S4C), and one of the three identified mutations occurred three times independently (Table S4).

The cell wall hydrolase N-acetylmuramoyl-L-alanine amidase (AmiA) has been implicated in septum digestion and peptidoglycan turnover (29). We identified mutations in the *amiA* gene in six independent lineages. Five of the six mutations were either premature stop codons or nucleotide deletions (Fig. S4; Table S4), suggesting that the inactivation of this autolysin could help in peptidoglycan stability in the resistant mutants.

The enterococcal serine/threonine kinase and its cognate phosphatase [*stp*(*ireK*)/*stk*(*ireP*)] play a central role in resistance to antibiotics, mostly cephalosporins, and other cell wall stresses (30). Resistance is mediated by IreB phosphorylation. The lack of phosphorylation in IreB either by impairing substitutions or by the absence of IreK results in reduced cephalosporin resistance. To turn off the signaling pathway, the cognate phosphatase, IreP, dephosphorylates IreK (31, 32). We identified mutations in *ireP* and *ireB* in six of the sequenced resistant lineages (Table S4). Our data suggest that the identified mutations maintain IreB constantly phosphorylated or active, either by *ireP* inactivating mutations or by mutations that prevent IreB dephosphorylation.

The essential two-component system *walKR* controls cell wall metabolism in Firmicutes (33, 34) by the homeostatic regulation of the cell wall hydrolases required for peptidoglycan expansion during growth coordinating both processes (35). Mutations in the WalKR two-component system were identified in 6 of 10 sequenced lineages. In the lineages derived from ATCC 29212, JH2-2, and D32, the mutations occurred in the sensor histidine kinase gene *walK*. The only sequenced lineage derived from a susceptible background that did not acquire *walK/R* mutations was JL1Amp (JH2-2), and this lineage did not become ampicillin resistant, suggesting that *walK* is important in the acquisition of ampicillin resistance. All WalK mutations were amino acid substitutions, and the majority occurred early during the experiment. In LSL2Imi (LS4828), the mutation occurred in the *yycl* response regulator, which controls the activation/deactivation of the *walKR* system (Fig. 4D; Table S4).

**Fig 5:**
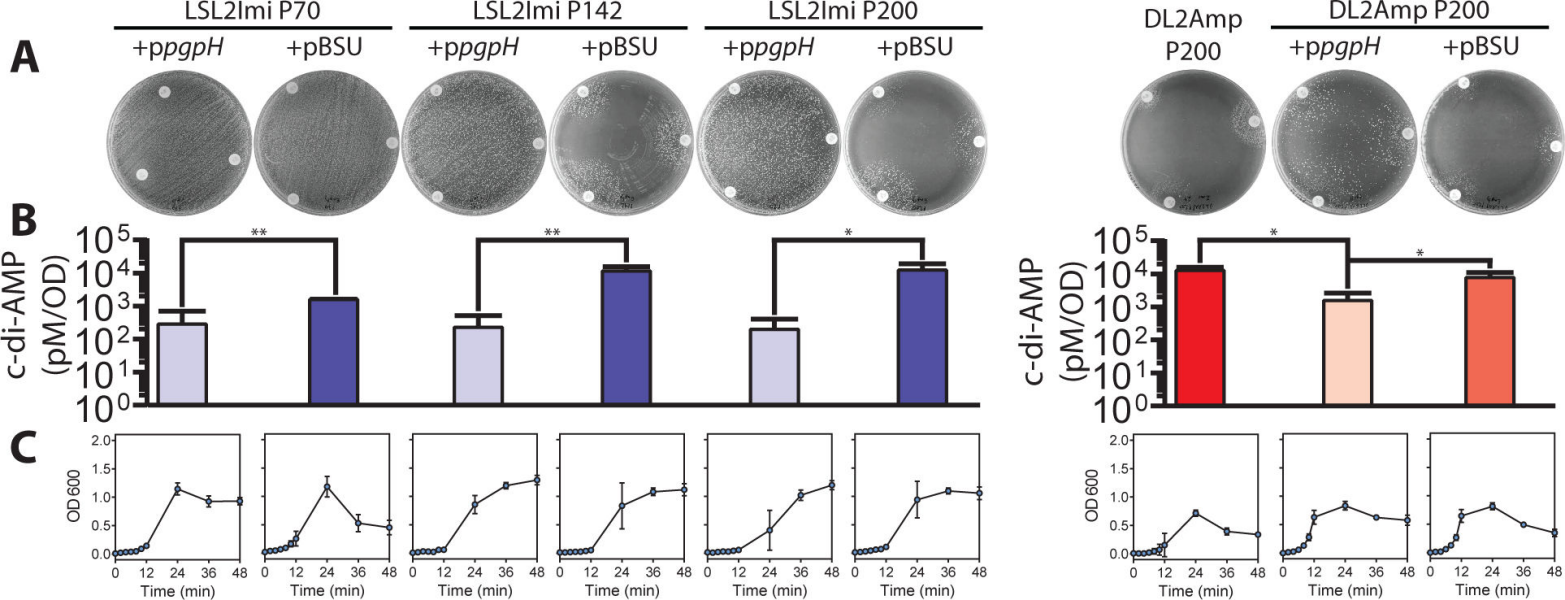
The presence of a functional copy of *pgpH* inhibits the β-lactam-dependent phenotype by decreasing the level of c-di-AMP. Passages 70 (without *pgpH* mutation), 142, and 200 (with a premature STOP codon at *pgpH*) from lineage LSL2imi and passage 200 from DL2Amp were transformed with the plasmid p*pgpH* (full-length *pgpH* with a constitutive promoter) or pBSU (negative control). (**A**) Disc diffusion assay with the complemented passages using ampicillin (bottom right of the plate, AM—10 µg), imipenem (bottom left of the plate, IPM—10 µg), and penicillin (top of the plate, P—10 µg) discs. The petri dishes show the complete inhibition (LSL2imi) or the softening (DL2Amp) of the β-lactam-dependent phenotype when the passages express the full-length version of *pgpH*. (**B**) C-di-AMP levels (pM/OD) in the complemented passages and their corresponding controls. Error bars indicate the standard deviation of the mean of three independent experiments. *P  ≤  0.05; **P  ≤  0.01. (**C**) Growth profile of the complemented passages determined by optical density (OD600).

We identified mutations in other genes involved in cell wall homeostasis that were lineage or strain specific and not shared between the different resistant mutants as shown in the supplemental data set.

### Mutations in the c-di-AMP pathway

We identified mutations in *cdaA, pgpH,* and *gdpP* in our evolved lineages (Table S5). We identified *cdaA* mutations (amino acid substitutions) in three lineages (one derived from ATCC 29212 and two from JH2-2), the mutations were all located within the regions coding for the transmembrane domains. No mutations were identified within the catalytic deadenylate cyclase domain (Fig. S5A). The *cdaA* mutations occurred relatively early during the LTEE between passages 14 and 70. We also identified a deletion in the putative *cdaA* regulator in one lineage derived from JH2-2. Additionally, we identified mutations in *pgpH* in five lineages from the four different backgrounds (Table S5) and *gdpP* mutations in two lineages derived from JH2-2. The mutations in the phosphodiesterases were either premature stop codons or amino acid substitutions in the catalytic domain (Fig. S5B and C; Table S5).

### c-di-AMP is elevated in resistant and dependent lineages

**Fig 6:**
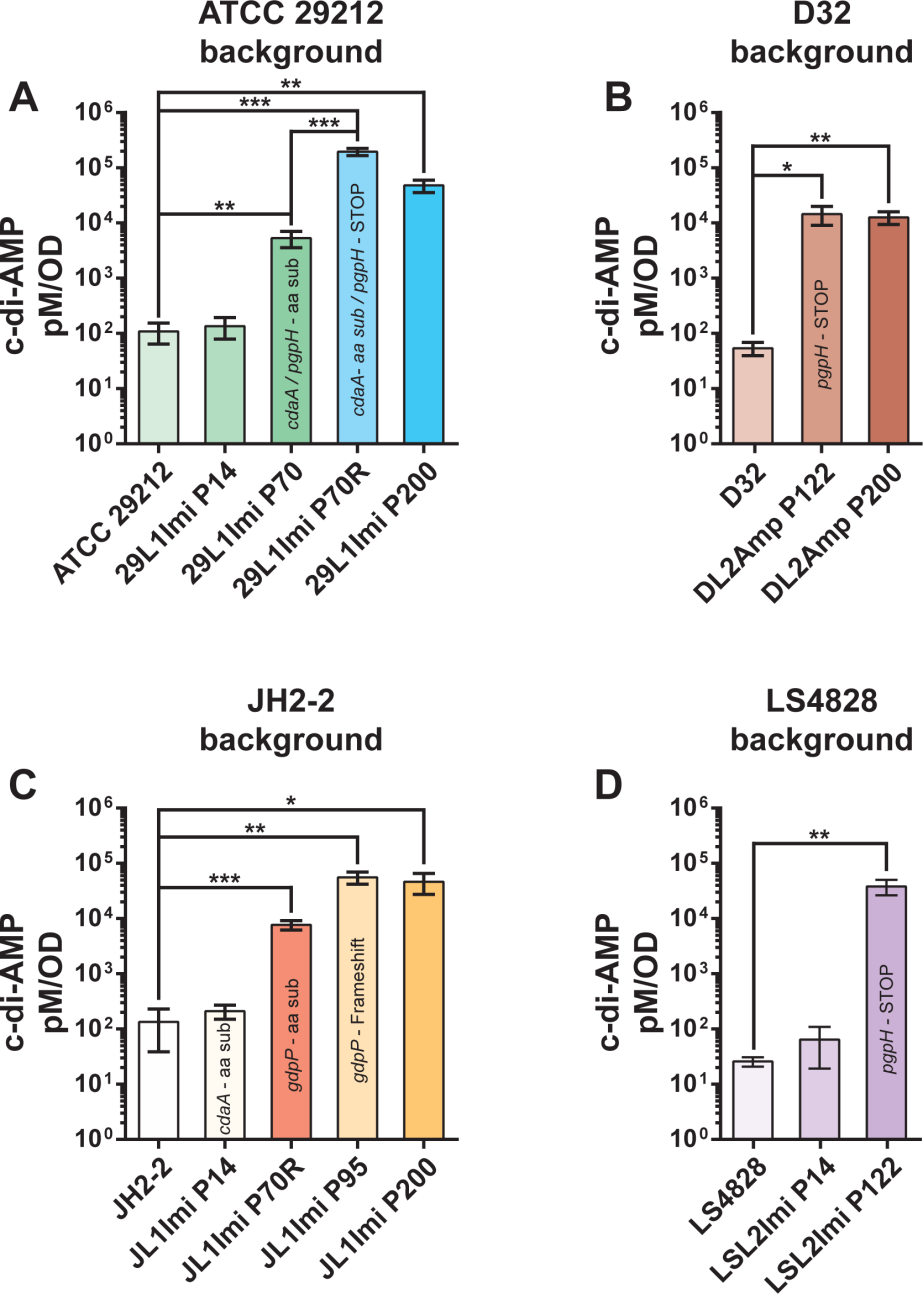
c-di-AMP quantification. The graphs show c-di-AMP levels in four different lineages carrying *cdaA* or *pgpH/gdpP* mutations. The passage in which a particular mutation was detected is labeled with the mutated gene. (**A**) ATCC 29212-derived lineages 29L1Imi Original and Replica. *cdaA* and *pgpH* mutations were acquired before the split of the original and replica plates. (**B**) D32-derived lineage DL2Amp, selected with ampicillin. This lineage acquired an inactivating pgpH mutation at P122, significantly increasing c-di-AMP concentrations. (**C**) JH2-2-derived lineage JL1Imi, selected with imipenem. This lineage acquired a *cdaA* mutation at P14 that did not produce significant changes in c-di-AMP concentrations and independent *gdpP* mutations (P70R and P95O) that both correlate with a significant increase in c-di-AMP intracellular concentrations. (**D**) LS4828-derived lineage LSL2Imi acquired an inactivating pgpH mutation at P122 with a significant increase in c-di-AMP levels. *P ≤ 0.05; **P ≤ 0.01.

Due to the ubiquity of mutations in c-di-AMP-regulating genes during the selection process in the LTEE, we were interested in determining c-di-AMP levels at different time points in β-lactam-resistant lineages. We performed c-di-AMP quantification by competitive ELISA. We found that *cdaA* mutations had little to no impact on c-di-AMP levels; however, lineages with mutations in either *pgpH* or *gdpP* reached very high levels of intracellular c-di-AMP (Fig. 6). *Trans*-complementation studies with a plasmid carrying a wild-type copy of *pgpH* under a constitutive promoter significantly reduced c-di-AMP levels in two resistant and dependent lineages with *pgpH* mutations, improved growth, and eliminated dependence without modifying the resistance to β-lactams (Fig. 5). Our results have shown that all but one lineage with *pgpH* or *gdpP* mutations developed β-lactam dependence and that all dependent lineages sequenced carried *pgpH* or *gdpP* mutations, indicating that mutations in one of the two c-di-AMP degrading enzymes are necessary but not sufficient for the β-lactam-dependent phenotype.

### Summary of genetic changes in β-lactam-resistant lineages

The acquisition of β-lactam resistance documented throughout the adaptive evolution of our selected lineages suggests a role of distinct mutations that were acquired in more than one genome and that correlated with changes in the MICs. Other mutations may not exhibit a direct role in β-lactam resistance but may be adaptations to restore fitness. To gain a deeper insight into the evolutionary trajectory of β-lactam adaptation, we correlated various aspects observed throughout the evolution of four selected lineages. The aspects considered were increasing antibiotic concentration, MIC values, cumulative mutated genes, intracellular concentration of c-di-AMP, development of antibiotic dependence, and growth.

**Fig 7:**
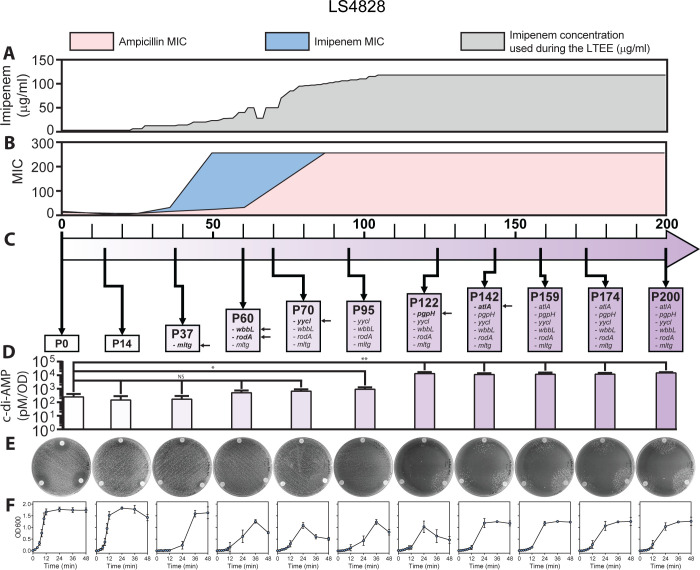
Timeline of the gradual adaptations to imipenem of LSL2Imi during the LTEE. (**A**) Gradual increment in imipenem concentration (µg/mL, gray) during the LTEE. (**B**) Ampicillin (pink) and imipenem (blue) MICs. (**C**) Mutated genes with a putative role in cell wall homeostasis (the squares correspond to the first time each mutation was identified). (**D**) c-di-AMP levels (pM/OD), results from three independent experiments. A Student’s *t*-test applying a *P* > 0.05 was used to compare passage 0 (parental strain) with the rest of the passages. *P ≤ 0.05; **P ≤ 0.01; ***P ≤ 0.001; and ****P ≤ 0.0001. (**E**) Disc diffusion assay with ampicillin (top of the plate, AM—10 µg), imipenem (bottom right of the plate, IPM—10 µg), and penicillin (bottom left of the plate, P—10 µg). The petri dishes show the transition from resistance to β-lactam dependence along the experiment. (**F**) Growth curves from three independent experiments.

In the β-lactam-resistant LS4828-derived lineage LSL2Imi, the first mutation identified was a premature stop codon in *mltg* at passage 37, eliminating the last 57 amino acids that correspond to part of the putative active domain. *mltg* codes for a putative endolytic murein transglycosylase/peptidoglycan muramidase. We did not observe changes in the MICs at this time point; however, we observed decreased fitness, determined as a delay in growth. Between passages 37 and 60, imipenem MIC increased from 8 to >256 µg/mL, and the lineage acquired mutations in *wbbl* and *rodA*. Neither *wbbl* nor *rodA* have been previously associated with antibiotic resistance in *Enterococcus*, but the role of RodA in cell wall homeostasis supports further studies. We observed cell lysis during the stationary phase starting at passage 60 and continuing until passage 122; lysis ended at passage 142 in association with a null mutation in the major autolysin gene *altA*. In passage 70, there was a single mutation in the WalK regulator gene *yycl*; we did not observe major changes in MICs or growth. Passage 95 had an increase in the ampicillin MIC to >256; however, no new mutations were identified at this time point, suggesting that the cumulative effect of previous mutations helps this lineage to withstand higher and higher ampicillin concentrations. The presence of a null mutation in the *pgpH* gene at passage 122 correlated with a statistically significant increase in intracellular c-di-AMP, compared to P95, and correlated with the emergence of β-lactam dependency (Fig. 7).

**Fig 8:**
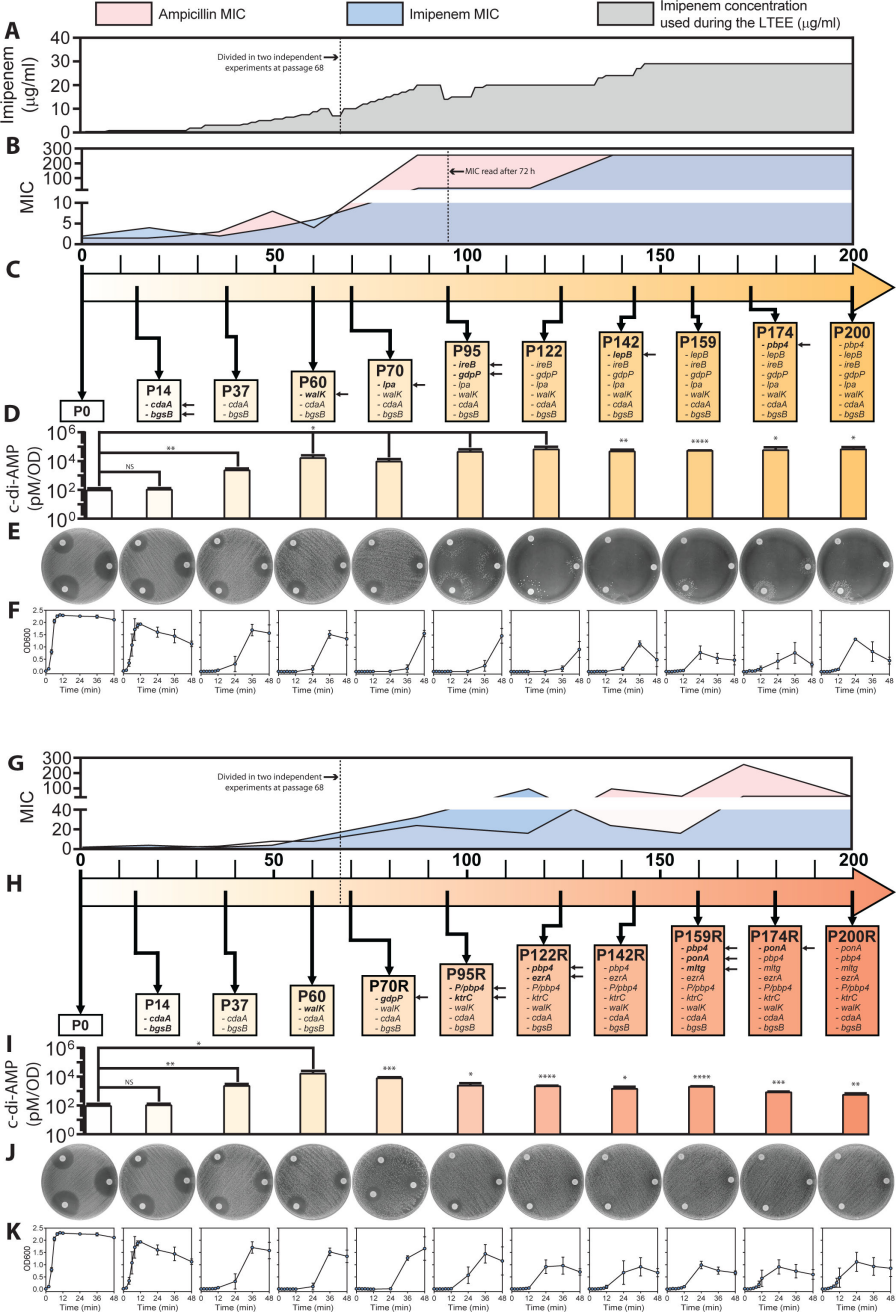
Timeline of the gradual adaptations to imipenem of JL1Imi during the LTEE in original and replica cultures. (**A**) Gradual increment in the imipenem concentration (µg/mL, gray) during the LTEE. (**B**) MICs of ampicillin (pink) and imipenem (blue). The MICs were read from passages 95 and up to 200 at 72 h because of the growing delay of those passages in the original culture. (**C**) Mutated genes with a putative role in resistance or fitness restoration. The mutated genes that appeared for the first time in specific passages are pointed out by arrows. (**D**) Levels of c-di-AMP (pM/OD) throughout the experiment. Error bars indicate the standard deviation of the mean of three independent experiments. A Student *t*-test applying a *P* > 0.05 was used to compare passage 0 (parental strain) with the rest of the passages. *P ≤ 0.05; **P ≤ 0.01; ***P  ≤  0.001; and ****P ≤ 0.0001. (**E**) Disc diffusion assay with ampicillin (bottom right of the plate, AM—10 µg), imipenem (bottom left of the plate, IPM—10 µg), and penicillin (top left of the plate, P—10 µg). The petri dishes show the transition of the passages from a resistant phenotype to a β-lactam-dependent phenotype across the experiment. (**F**) Growth curves determined by optical density (OD600). The lines in the graphs represent the mean of three independent experiments. Error bars indicate the standard deviation. The same aspects were measured in the replica culture. (**G**) MICs. (**H**) Mutated genes. (**I**) c-di-AMP levels. (**J**) Disc diffusion assay. (**K**) Growth curves. The images for passage 0 to passage 60 in panels Eand J and panels F and K are the same because they were taken before the serial dilutions were split. They have been utilized in both panels for ease of comparison.

In the JH2-2-derived lineage JL1Imi, the first two mutations were detected early during the experiment when the imipenem concentration used for selection was still very low, at passage 14 in *cdaA* and *bgsB* (1,2-diacylglycerol 3-glucosyltransferase), correlating with a delay in growth. Growth did not recover in this lineage, and the growth curves show growth only after 24 h or later, suggesting that the population recovery observed is due to new mutations acquired during the growth curve experiment (Fig. 8F); we currently do not have sequencing data for these samples. Mutations in *walK* and *lpa* (lipoamidase) at passages 60 and 70, respectively, were associated with a moderate increase in MIC for ampicillin and imipenem. At passage 70, we observed a sharp decrease in fitness, measured as growth. As we progressively increased the imipenem concentration, we documented *ireB* and *gdpP* mutations at passage 95 in the original culture (Fig. 6A through F). These mutations coincided with the development of β-lactam dependence and an inability to obtain MIC readings at 24 h due to delayed growth. The presence of a mutation in *lepB* (a signal peptidase) at passage 142 correlated with better growth. This lineage acquired a *pbp4* mutation later in the experiment at passage 174.

The replica culture (Fig. 6G through K) once split from the original culture evolved a different trajectory. At passage 70, it acquired an independent mutation in *gdpP* (amino acid substitution, compared to premature stop codon), which coincided with a modest increase in MIC and a significant increase in c-di-AMP concentrations compared to previous passages; however, c-di-AMP levels were not as high as in lineages carrying premature stop codons. Interestingly, this lineage did not develop dependence. Additionally, this lineage acquired four independent mutations in *pbp4* (detected at passages 95R, 122R, 159R, and 200R). Ampicillin MICs increased at passages 95R, 142–149R, and 174R. Imipenem MICs increased at passages 95R and 122R. In addition to *pbp4* mutations, we identified two mutations in *ponA* (passages 159R and 174R), and a convergent mutation in *lpa*.

In the ATCC 29212-derived lineages, 29L1ImiO and 29L1ImiR followed different trajectories to acquire β-lactam resistance and develop antibiotic dependence. 29L1ImiR lineage acquired resistance to ampicillin and imipenem at passage 60 and passage 70R, respectively. At this time, we identified mutations in *cdaA, pgpH, pbp4, ireB,* and *walK* and observed an initially weak dependent phenotype at passage 70R. A mutation in *ponA* at passage 95R coincided with a further increase in MICs. Two additional mutations in *pbp4* (passages 122R and 174R) and a mutation in *amiA* (passage 174R) seemed to not have a further impact on the MICs; however, MIC testing in this lineage shows fluctuating results and high variability.

The dependent phenotype is highly variable and does not always correlate temporarily with the acquisition of mutations in the c-di-AMP pathway or with the peak concentration of this metabolite, as exemplified in the differences observed between the original and replica cultures of 29L1Imi. 29L1ImiO acquired mutations in *cdaA* and *pgpH* (independently of 29L1ImiR) at passage 70 but developed a dependent phenotype only at passage 95 and later lost dependency at passage 175.

DL2Amp was derived from D32 under selection with ampicillin. Resistance to ampicillin was acquired at passage 95 when it had accumulated *walK, pbp2B,* and *ireP* mutations, resistance to imipenem was detected at passage 122, coincidental with a *pgpH* mutation and increased c-di-AMP levels. A β-lactam-dependent phenotype was evident only in passages 174 and 200.

Mutations in *pgpH* and elevated levels of c-di-AMP seem to be necessary for the development of β-lactam dependence.

## DISCUSSION

The acquisition of β-lactam resistance is a complex phenomenon, accompanied by evolutionary trade-offs. The results presented in this paper confirm the capacity of *E. faecalis* strains to acquire markedly elevated levels of resistance when confronted with persistent selective pressure from β-lactam antibiotics. We were able to achieve levels of resistance considerably greater than obtained in previous reports (9, 36). The use of four distinct genetic backgrounds and the splitting of these strains into different lineages allowed us to look for convergent genes and pathways involved in resistance, of which we found several. While clinical isolates demonstrating MICs to ampicillin and imipenem ranging between 12 and 32 µg/mL have been documented (9, 36), high MICs of 256 µg/mL to both ampicillin and imipenem are uncommon among *E. faecalis* isolates. Nevertheless, there is a report of some *E. faecalis* isolates from patients that reached an MIC for imipenem of >256 µg/mL, although the genetic or metabolic determinants involved remain elusive (37). We did not identify additional reports of either clinical isolates or laboratory-evolved lineages of *E. faecalis* achieving the higher MICs observed in our study (>256 µg/mL). Enterococci typically display resistance to most β-lactams, exhibiting relative susceptibilities to certain members of the penicillin group, such as ampicillin, penicillin, and piperacillin (1). Resistance to ampicillin and imipenem in *E. faecalis* is closely linked to the overexpression of low-affinity class B Pbp4 induced by mutations in the promoter region. We and others had previously reported promoter deletions leading to *pbp4* overexpression in both clinical and laboratory strains (9, 38–40), suggesting that the mutations identified in the current work will have a similar effect on *pbp4* transcription.

Amino acid substitutions have also been implicated in ampicillin/imipenem resistance in *E. faecalis* (9, 36, 39, 41–43), but the resulting resistance levels are not as pronounced as those reported here. Hence, we consider that the exceptionally high resistance levels observed are attributed to the sum of factors in addition to Pbp4.

Our data indicate that resistance in virtually all strains emerges more easily to imipenem than to ampicillin, and it emerges more readily under imipenem selective pressure than under pressure from ampicillin, especially in the strains that were initially susceptible. These results are in line with what we and others have reported with the clinical use of amoxicillin or imipenem (9, 38). To our knowledge, there are no known differences in the effects of imipenem vs ampicillin on *E. faecalis* cells, such as differential reactivities of imipenem and ampicillin with the PBPs that could explain the differences observed in our study. Further work would provide interesting insights into this observation.

As mentioned before, β-lactam resistance in *E. faecalis* has most frequently been tied to changes within Pbp4. These changes have additionally involved amino acid substitutions, many of which are assumed to reduce affinity for the antibiotic, but only a few of which have been documented to do so (17, 28, 36, 39). We observed seven different amino acid substitutions in Pbp4 over the course of the experiment, with two appearing in more than one strain. While we do not have experimental evidence that these changes confer resistance, they are all in proximity to the active site (W. Peti, personal communication), so they may either alone or in combination confer reduced susceptibility to ampicillin, imipenem, or both.

It is conceivable that prolonged and consistent exposure to β-lactams may induce a recurring pattern of mutations within specific genes associated with resistance. It is also noteworthy that strain LS4828, a strain clinically resistant to β-lactams, underwent no further Ppb4 substitutions yet achieved high levels of resistance in the shortest time compared with the other three susceptible backgrounds. The increase of ampicillin and imipenem MICs in LS4828-evolved lineages cannot be attributed to *pbp4* mutations. One gene that is interesting in the context of β-lactam resistance is *mltG*, coding for a putative peptidoglycan muramidase essential for cell elongation in streptococci (44). Additional mutations occur in RodA, a glycosyltransferase that partners with PBP transpeptidases to make peptidoglycan during cellular elongation and division (45), and WbbL (L-rhamnosyltransferase of unknown function in *E. faecalis* that has analogs in Gram-negative bacteria that are involved in lipopolysaccharide biosynthesis and is important for the synthesis of O-antigen) (46), which could be contributing to further increases in the imipenem MICs. Although the interaction between MltG and RodA has not been investigated in *E. faecalis*, this interaction was reported in *Escherichia coli* (47). Additionally, a model of how these proteins interact was proposed in *Streptococcus pneumoniae*, in which MltG and other amidases open the peptidoglycan layer, letting RodA interact with a PBP to add new peptidoglycans to the layer, inducing the elongation of the cell (45). Another interesting gene is *yycl*, a regulator of the WalR/WalK two-component system, which has been shown to be involved in cell wall synthesis and antimicrobial susceptibility in different species, including *Staphylococcus aureus* and *Bacillus subtilis* (35, 48–50). In *S. aureus,* the disruption of *yycH* and *yycI* genes led to a downregulation of the WalKR regulon, including reduced expression of the autolysin genes *atlA* and *sle1,* inducing impaired cell wall turnover and reduced vancomycin susceptibility (51). In contrast, *B. subtilis* cells lacking *yycH* or *yycl* showed stronger transcription of autolysins, suggesting that the active genes lead to a reduction of WalK activity (52–54). Our data suggest that in *E. faecalis,* the disruption of *yycl* induces activation of the WalKR regulon and that compensatory mutations such as the frameshift observed in *atlA* gene compensate an overactive WalKR and help keep cell wall turnover in check, improving growth rate at 12 h. Further studies are necessary to validate the role of the mutated genes.

Our data indicate that there is strain specificity in the types of phenotypic resistance and genetic changes observed when the strains are under selective antimicrobial pressure. The pattern of mutations observed on different strains selected under ampicillin or imipenem pressure is more similar within the strains than between strains. A similar observation was made by Card and colleagues (21) in *E. coli*, who concluded that strain genotype can affect both the genotypic and phenotypic pathways to resistance.

An intriguing finding of our study is that there appears to be a final common phenotypic characteristic of all strains—antibiotic dependence. This is a lethal phenotype in the absence of antibiotics; however, eventually, progeny emerge that can regain growth in the absence of antibiotics. Loss of dependence does not affect β-lactam resistance. The evolutionary advantage of β-lactam dependence vs β-lactam resistance could rely on the inactivation by the antibiotic of other PBPs letting a highly resistant PBP4 take over the synthesis of cell wall. β-lactam-dependent lineages do not overexpress PBP4 upon exposure to the antibiotics. The evolution of β-lactam dependence in our selected lineages was associated with inactivating mutations in one of the two genes coding for the c-di-AMP phosphodiesterases. Consistent with the inactivating nature of these mutations, we were able to measure markedly increased quantities of c-di-AMP in mutant lineages. We confirmed the involvement of c-di-AMP levels in this phenotype by introducing a plasmid-encoded intact *pgpH*, maintaining β-lactam resistance, and losing the dependent phenotype. In *Staphylococcus aureus,* inactivating mutations have been associated with β-lactam tolerance and enhanced evolution of resistance both *in vitro* and in clinical strains (55, 56). Tolerance to β-lactams could be caused by a modulation in cell wall thickness (57); further studies are necessary to determine if a similar mechanism occurs in *E. faecalis* and how this leads to β-lactam dependence. C-di-AMP is an important second messenger in Gram-positive bacteria (58), and it has been implicated in virulence, susceptibility to cell envelope-targeting drugs, cell membrane-acting antibiotics, peptide and metabolite transport, osmotic regulation, and other fundamental cell functions (59). Homeostasis of c-di-AMP is of paramount importance since under many growth conditions, it is essential, but its accumulation is also toxic (60–62). *E. faecalis* codes for a single diadenylate cyclase (CdaA), which is responsible for c-di-AMP synthesis, and two phosphodiesterases in charge of c-di-AMP degradation (PgpH and GdpP) (58, 60–64). C-di-AMP is essential during normal growth conditions in *E. faecalis* (64).

While some mutations are clearly associated with resistance or dependence, it is plausible that others are more closely linked with fitness restoration after the tradeoff of antibiotic resistance. Mutations improving the fitness of resistant mutants can be epistatic, jointly contributing to adaptation under specific pressures (65). However, owing to the inherently stochastic dynamics of β-lactam resistance and fitness-related epistatic interactions, the data gathered in this study represent just a few of many possible evolutionary trajectories. These data may contribute toward uncovering patterns for predicting models that closely mirror the trajectory of *E. faecalis* evolution in response to β-lactam antibiotics. Consequently, more focused studies will be necessary in the future.

## MATERIALS AND METHODS

### Long-term evolution experiment

To obtain β-lactam-resistant lineages, four *E. faecalis* strains from different origins and with different initial susceptibilities (Table 1) were subjected to serial passaging in an LTEE. We exposed the four distinct *E. faecalis* strains to continuous and increasing ampicillin or imipenem selective pressure for a total of 6,000 generations (200 days). We started the LTEE from three founding colonies from each strain. Each founding colony was seeded into ampicillin, imipenem, or no antibiotic, establishing a total of 36 initial cultures: 12 were grown with ampicillin, 12 were grown with imipenem, and 12 were grown in broth alone. The antibiotic concentrations were increased gradually depending on the growth rate until the experiment reached 104 daily passages. After day 105, we kept the antibiotic concentration constant until day 200 because several lineages displayed very low growth rates. Samples were frozen every 10 passages for each culture (Fig. S1). The initial ampicillin and imipenem concentration was 1/4 MIC for each strain. The LTEE was carried out in BHI media (66). Cultures were passaged by 100-fold dilution (5 µL in 500 µL) every 24 h as reported by Lenski and Travisano in 1994 (67). The initial inoculum at the start of the experiment was OD_600_ ~0.001 (~7.00E+03 CFUs). Initially, when the antibiotic selection pressure was low, growth after 24 h in all plates was about 7.70E+09 CFUs/mL (OD_600_ 0.8), and each fresh well was inoculated with 7.70E+07 CFUs/mL (~3.85E+05 in 5 µL). As the LTEE progressed and the growth rate became more variable between the different lineages, the CFUs inoculated were also more variable; moreover, several lineages developed a clumpy phenotype that made accurate quantification complicated. Weekly sampling and storage were performed for each lineage. MICs to ampicillin and imipenem were determined every ~450 generations (15 days) by MIC strip tests on MHII agar plates (68).

### Genomic analysis

Whole-genome sequencing was performed by Psomagen (MD) or SeqCenter (PA). Read length was 151 reads with an average coverage of 2,000× (Psomagen) and 30× (SeqCenter). *De novo* genome assembly was performed with SPAdes version 3.12 (69), using the default parameters. Genome annotation was performed with Prokka (70). SPAdes and Prokka were run through the Brown University supercomputing cluster OSCAR. Variant calling was performed with Geneious 11.1.5 using default parameters for bacterial genomes. Additional variant calling analysis was carried out by SeqCenter using breseq version 0.35.4 (71).

### Intracellular cyclic di-AMP measurement by competitive ELISA

Cyclic di-AMP ELISA (enzyme-linked immunosorbent assay) Kit (Cayman Chemical) was used for the quantification of c-di-AMP. Briefly, the samples were assayed using a minimum of two dilutions in duplicate. Sample and standard volumes of 50 µL were added to the pre-coated goat anti-mouse IgG and pre-blocked microtiter plate followed by the addition of 50 µL of cyclic di-AMP-horseradish peroxidase (HRP) conjugate tracer and 50 µL of cyclic di-AMP monoclonal antibody. Appropriate controls according to the manufacturer’s directions were also added at this point. The plate was covered and incubated at room temperature on an orbital shaker for 2 h. The wells were emptied, washed five times with provided wash buffer with Tween 20, and filled with 175 µL of 3,3',5,5′-tetramethylbenzidine substrate solution plus 5 µL of the diluted tracer to the appropriate well. The plate was covered and incubated at room temperature for 30 min on the orbital shaker. The reaction was stopped with 75 µL of HRP stop solution, and the plate was read at 450 nm in a BioTek EPOCH2 microplate reader. The data were analyzed using a computer spreadsheet provided by Cayman Chemical. Using the spreadsheet, the standard curve data were linearized using a logit transformation, and the sample values were determined from the standard curve.

### Statistical analysis

Data from c-di-AMP measurements were analyzed with GraphPad Prism version 6.0 (GraphPad Software, San Diego, CA, USA). Data from three independent experiments were plotted, and a two-tailed Student’s *t*-test was used to compare each group vs each other. A *P* > 0.05 value was considered statistically significant.

## Data Availability

WGS data are available from NCBI with accession numbers PRJNA779716, JBGCWG000000000, JBGCWH000000000, and JBGCWF000000000. Variant calling analysis is available as Data sets S1, S2, S3, and S4. Protein accession numbers are provided in Table S6.

## SUPPLEMENTAL MATERIAL

Supplemental figures mbio.02897-24-s0001.pdf Fig. S1 to S5.

Tables S1 and S2 mbio.02897-24-s0002.xlsx Lineage identification key and sequenced genomes.

Table S3 mbio.02897-24-s0003.xlsx Disc diffusion assays.

Tables S4 and S5 mbio.02897-24-s0004.xlsx Mutations identified in genes related to cell wall synthesis and degradation.

Table S6 mbio.02897-24-s0005.xlsx Protein accession numbers.

Supplemental text mbio.02897-24-s0006.docx Supplemental methods and legends.

Supplemental data sets mbio.02897-24-s0007.xlsx Lists of mutations.

ASM does not own the copyrights to Supplemental Material that may be linked to, or accessed through, an article. The authors have granted ASM a non-exclusive, world-wide license to publish the Supplemental Material files. Please contact the corresponding author directly for reuse.

## References

[1] García-Solache M, Rice LB. 2019. The Enterococcus: a model of adaptability to its environment. Clin Microbiol Rev 32:e00058-18. doi:10.1128/CMR.00058-18 30700430 PMC6431128

[2] Weiner LM, Webb AK, Limbago B, Dudeck MA, Patel J, Kallen AJ, Edwards JR, Sievert DM. 2016. Antimicrobial-resistant pathogens associated with healthcare-associated infections: summary of data reported to the national healthcare safety network at the centers for disease control and prevention, 2011–2014. Infect Control Hosp Epidemiol 37:1288–1301. doi:10.1017/ice.2016.174 27573805 PMC6857725

[3] Sugden R, Kelly R, Davies S. 2016. Combatting antimicrobial resistance globally. Nat Microbiol 1:16187. doi:10.1038/nmicrobiol.2016.187 27670123

[4] Zimmer SM, Caliendo AM, Thigpen MC, Somani J. 2003. Failure of linezolid treatment for enterococcal endocarditis. Clin Infect Dis 37:e29–30. doi:10.1086/375877 12884185

[5] Piszczek J, Hutchinson J, Partlow E. 2015. Failure of combination therapy with daptomycin and synergistic ceftriaxone for enterococcal endocarditis. J Antimicrob Chemother 70:623–624. doi:10.1093/jac/dku381 25246438

[6] Kim D, Lee H, Yoon E-J, Hong JS, Shin JH, Uh Y, Shin KS, Shin JH, Kim YA, Park YS, Jeong SH. 2019. Prospective observational study of the clinical prognoses of patients with bloodstream infections caused by ampicillin-susceptible but penicillin-resistant Enterococcus faecalis. Antimicrob Agents Chemother 63:e00291–19. doi:10.1128/AAC.00291-19 31010856 PMC6591605

[7] Kristich CJ, Rice LB, Arias CA. 2014. Enterococcal infection—treatment and antibiotic resistance. In Gilmo MS, Clewell DB, Ike Y, Shankar N (ed), Enterococci: from commensals to leading causes of drug resistant infection. Massachusetts Eye and Ear Infirmary, Boston. 24649510

[8] Agudelo Higuita NI, Huycke MM. 2014. Enterococcal disease, epidemiology, and implications for treatment. In Gilmo MS, Clewell DB, Ike Y, Shankar N (ed), Enterococci: from commensals to leading causes of drug resistant infection. Massachusetts Eye and Ear Infirmary, Boston. 24649504

[9] Rice LB, Desbonnet C, Tait-Kamradt A, Garcia-Solache M, Lonks J, Moon TM, D’Andréa ÉD, Page R, Peti W. 2018. Structural and regulatory changes in PBP4 trigger decreased β-lactam susceptibility in Enterococcus faecalis. MBio 9:e00361-18. doi:10.1128/mBio.00361-18 29615500 PMC5885037

[10] López-Luis BA, Sifuentes-Osornio J, Lambraño-Castillo D, Ortiz-Brizuela E, Ramírez-Fontes A, Tovar-Calderón YE, Leal-Vega FJ, Bobadilla-Del-Valle M, Ponce-de-León A. 2021. Risk factors and outcomes associated with vancomycin-resistant Enterococcus faecium and ampicillin-resistant Enterococcus faecalis bacteraemia: a 10-year study in A tertiary-care centre in Mexico City. J Glob Antimicrob Resist 24:198–204. doi:10.1016/j.jgar.2020.12.005 33359937

[11] Mousavi SH, Peeri-Doghaheh H, Mohammadi-Ghalehbin B, Teimourpour R, Maleki D, Khademi F, Arzanlou M. 2020. High-level resistance to aminoglycosides and ampicillin among clinical isolates of Enterococcus species in an Iranian referral hospital. Iran J Microbiol 12:319–324. doi:10.18502/ijm.v12i4.3935 32994903 PMC7502148

[12] Esmail MAM, Abdulghany HM, Khairy RM. 2019. Prevalence of multidrug-resistant Enterococcus faecalis in hospital-acquired surgical wound infections and bacteremia: concomitant analysis of antimicrobial resistance genes. Infect Dis (Auckl) 12:1178633719882929. doi:10.1177/1178633719882929 31662606 PMC6796195

[13] Eron LJ. 1985. Imipenem/cilastatin therapy of bacteremia. Am J Med 78:95–99. doi:10.1016/0002-9343(85)90108-1 3859221

[14] Donabedian H, Freimer EH. 1985. Pathogenesis and treatment of endocarditis. Am J Med 78:127–133. doi:10.1016/0002-9343(85)90115-9 3890533

[15] Clissold SP, Todd PA, Campoli-Richards DM. 1987. Imipenem/Cilastatin. Drugs (Abingdon Engl) 33:183–241. doi:10.2165/00003495-198733030-00001 3552595

[16] Simonsen GS, Småbrekke L, Monnet DL, Sørensen TL, Møller JK, Kristinsson KG, Lagerqvist-Widh A, Torell E, Digranes A, Harthug S, Sundsfjord A. 2003. Prevalence of resistance to ampicillin, gentamicin and vancomycin in Enterococcus faecalis and Enterococcus faecium isolates from clinical specimens and use of antimicrobials in five Nordic hospitals. J Antimicrob Chemother 51:323–331. doi:10.1093/jac/dkg052 12562698

[17] Conceição N, da Silva LEP, Darini AL da C, Pitondo-Silva A, de Oliveira AG. 2014. Penicillin-resistant, ampicillin-susceptible Enterococcus faecalis of hospital origin: pbp4 gene polymorphism and genetic diversity. Infect Genet Evol 28:289–295. doi:10.1016/j.meegid.2014.10.018 25445645

[18] Andersson DI, Hughes D. 2010. Antibiotic resistance and its cost: is it possible to reverse resistance? Nat Rev Microbiol 8:260–271. doi:10.1038/nrmicro2319 20208551

[19] Vogwill T, MacLean RC. 2015. The genetic basis of the fitness costs of antimicrobial resistance: a meta-analysis approach. Evol Appl 8:284–295. doi:10.1111/eva.12202 25861386 PMC4380922

[20] Andersson DI, Levin BR. 1999. The biological cost of antibiotic resistance. Curr Opin Microbiol 2:489–493. doi:10.1016/s1369-5274(99)00005-3 10508723

[21] Card KJ, Thomas MD, Graves JL, Barrick JE, Lenski RE. 2021. Genomic evolution of antibiotic resistance is contingent on genetic background following a long-term experiment with Escherichia coli. Proc Natl Acad Sci U S A 118:e2016886118. doi:10.1073/pnas.2016886118 33441451 PMC7865137

[22] Card KJ, LaBar T, Gomez JB, Lenski RE. 2019. Historical contingency in the evolution of antibiotic resistance after decades of relaxed selection. PLoS Biol 17:e3000397. doi:10.1371/journal.pbio.3000397 31644535 PMC6827916

[23] Andrewes FW, Horder TJ. 1906. A study of the streptococci pathogenic for man. Lancet 168:708–713. doi:10.1016/S0140-6736(01)31538-6

[24] Jacob AE, Hobbs SJ. 1974. Conjugal transfer of plasmid-borne multiple antibiotic resistance in Streptococcus faecalis var. zymogenes. J Bacteriol 117:360–372. doi:10.1128/jb.117.2.360-372.1974 4204433 PMC285522

[25] Minogue TD, Daligault HE, Davenport KW, Broomall SM, Bruce DC, Chain PS, Coyne SR, Chertkov O, Freitas T, Gibbons HS, Jaissle J, Koroleva GI, Ladner JT, Palacios GF, Rosenzweig CN, Xu Y, Johnson SL. 2014. Complete genome assembly of Enterococcus faecalis 29212, a laboratory reference strain. Genome Announc 2:e00968-14. doi:10.1128/genomeA.00968-14 25291775 PMC4175211

[26] Shankar N, Baghdayan AS, Willems R, Hammerum AM, Jensen LB. 2006. Presence of pathogenicity island genes in Enterococcus faecalis isolates from pigs in Denmark. J Clin Microbiol 44:4200–4203. doi:10.1128/JCM.01218-06 16957034 PMC1698369

[27] Lieberman MT, Van Tyne D, Dzink-Fox J, Ma EJ, Gilmore MS, Fox JG. 2018. Long-term colonization dynamics of Enterococcus faecalis in implanted devices in research macaques. Appl Environ Microbiol 84:e01336-18. doi:10.1128/AEM.01336-18 30006402 PMC6121975

[28] Tran TC, Simar SR, Egge SL, Atterstrom R, Dinh A, Hanson BM, Contreras G, Zervos M, Abbo LM, Shimose L, Shelburne SA, Arias CA, Miller WR. 2022. 128. association of PBP4 variants and β-lactam susceptibility in Enterococcus faecalis. Open Forum Infect Dis 9. doi:10.1093/ofid/ofac492.206

[29] Mesnage S, Chau F, Dubost L, Arthur M. 2008. Role of N-acetylglucosaminidase and N-acetylmuramidase activities in Enterococcus faecalis peptidoglycan metabolism. J Biol Chem 283:19845–19853. doi:10.1074/jbc.M802323200 18490448

[30] Kristich CJ, Wells CL, Dunny GM. 2007. A eukaryotic-type Ser/Thr kinase in Enterococcus faecalis mediates antimicrobial resistance and intestinal persistence. Proc Natl Acad Sci U S A 104:3508–3513. doi:10.1073/pnas.0608742104 17360674 PMC1805595

[31] Djorić D, Minton NE, Kristich CJ. 2021. The enterococcal PASTA kinase: a sentinel for cell envelope stress. Mol Oral Microbiol 36:132–144. doi:10.1111/omi.12313 32945615 PMC7969467

[32] Kristich CJ, Little JL, Hall CL, Hoff JS. 2011. Reciprocal regulation of cephalosporin resistance in Enterococcus faecalis. mBio 2:e00199-11. doi:10.1128/mBio.00199-11 22045988 PMC3202758

[33] Dubrac S, Bisicchia P, Devine KM, Msadek T. 2008. A matter of life and death: cell wall homeostasis and the WalKR (YycGF) essential signal transduction pathway. Mol Microbiol 70:1307–1322. doi:10.1111/j.1365-2958.2008.06483.x 19019149

[34] Takada H, Yoshikawa H. 2018. Essentiality and function of WalK/WalR two-component system: the past, present, and future of research. Biosci Biotechnol Biochem 82:741–751. doi:10.1080/09168451.2018.1444466 29514560

[35] Dobihal GS, Flores-Kim J, Roney IJ, Wang X, Rudner DZ. 2022. The WalR-WalK signaling pathway modulates the activities of both CwlO and LytE through control of the peptidoglycan deacetylase PdaC in Bacillus subtilis. J Bacteriol 204:e0053321. doi:10.1128/JB.00533-21 34871030 PMC8846395

[36] Ono S, Muratani T, Matsumoto T. 2005. Mechanisms of resistance to imipenem and ampicillin in Enterococcus faecalis. Antimicrob Agents Chemother 49:2954–2958. doi:10.1128/AAC.49.7.2954-2958.2005 15980374 PMC1168717

[37] Schouten MA, Voss A, Hoogkamp-Korstanje JA. 1999. Antimicrobial susceptibility patterns of enterococci causing infections in Europe. The European VRE study group. Antimicrob Agents Chemother 43:2542–2546. doi:10.1128/AAC.43.10.2542 10508041 PMC89517

[38] Van Tyne D, Manson AL, Huycke MM, Karanicolas J, Earl AM, Gilmore MS. 2019. Impact of antibiotic treatment and host innate immune pressure on enterococcal adaptation in the human bloodstream. Sci Transl Med 11:eaat8418. doi:10.1126/scitranslmed.aat8418 30971455 PMC6681809

[39] Lazzaro LM, Cassisi M, Stefani S, Campanile F. 2021. Impact of PBP4 alterations on β-lactam resistance and ceftobiprole non-susceptibility among Enterococcus faecalis clinical isolates. Front Cell Infect Microbiol 11:816657. doi:10.3389/fcimb.2021.816657 35127564 PMC8811369

[40] Conti P, Lazzaro LM, Longo F, Lenzo F, Giardina A, Fortuna SA, Stefani S, Campanile F. 2024. Unveiling the relationship between ceftobiprole and high-molecular-mass (HMM) penicillin-binding proteins (PBPs) in Enterococcus faecalis. Antibiotics (Basel) 13:65. doi:10.3390/antibiotics13010065 38247624 PMC10812503

[41] Gawryszewska I, Żabicka D, Hryniewicz W, Sadowy E. 2021. Penicillin-resistant, ampicillin-susceptible Enterococcus faecalis in Polish hospitals. Microb Drug Resist 27:291–300. doi:10.1089/mdr.2019.0504 32640911

[42] Infante VHP, Conceição N, de Oliveira AG, Darini AL da C. 2016. Evaluation of polymorphisms in pbp4 gene and genetic diversity in penicillin-resistant, ampicillin-susceptible Enterococcus faecalis from hospitals in different states in Brazil. FEMS Microbiol Lett 363:fnw044. doi:10.1093/femsle/fnw044 26903013

[43] Westbrook KJ, Chilambi GS, Nordstrom HR, Iovleva A, Shah NH, Jones CE, Kline EG, Doi Y, Shields RK, Tyne DV. 2021. Differential ampicillin/ceftriaxone susceptibility among diverse Enterococcus faecalis from infective endocarditis. bioRxiv. doi:10.1101/2021.06.07.447474:2021.06.07.447474 PMC10984950 38334390

[44] Tsui H-CT, Zheng JJ, Magallon AN, Ryan JD, Yunck R, Rued BE, Bernhardt TG, Winkler ME. 2016. Suppression of a deletion mutation in the gene encoding essential PBP2b reveals a new lytic transglycosylase involved in peripheral peptidoglycan synthesis in Streptococcus pneumoniae D39. Mol Microbiol 100:1039–1065. doi:10.1111/mmi.13366 26933838 PMC5063045

[45] Winther AR, Kjos M, Herigstad ML, Håvarstein LS, Straume D. 2021. EloR interacts with the lytic transglycosylase MltG at midcell in Streptococcus pneumoniae R6. J Bacteriol 203:e00691-20. doi:10.1128/JB.00691-20 33558392 PMC8092159

[46] Thänert R, Choi J, Reske KA, Hink T, Thänert A, Wallace MA, Wang B, Seiler S, Cass C, Bost MH, Struttmann EL, Iqbal ZH, Sax SR, Fraser VJ, Baker AW, Foy KR, Williams B, Xu B, Capocci-Tolomeo P, Lautenbach E, Burnham C-AD, Dubberke ER, Kwon JH, Dantas G, CDC Prevention Epicenters Program. 2022. Persisting uropathogenic Escherichia coli lineages show signatures of niche-specific within-host adaptation mediated by mobile genetic elements. Cell Host Microbe 30:1034–1047. doi:10.1016/j.chom.2022.04.008 35545083 PMC10365138

[47] Bohrhunter JL, Rohs PDA, Torres G, Yunck R, Bernhardt TG. 2021. MltG activity antagonizes cell wall synthesis by both types of peptidoglycan polymerases in Escherichia coli. Mol Microbiol 115:1170–1180. doi:10.1111/mmi.14660 33278861 PMC9020800

[48] Gajdiss M, Monk IR, Bertsche U, Kienemund J, Funk T, Dietrich A, Hort M, Sib E, Stinear TP, Bierbaum G. 2020. YycH and YycI regulate expression of Staphylococcus aureus autolysins by activation of WalRK phosphorylation. Microorganisms 8:870. doi:10.3390/microorganisms8060870 32526915 PMC7355866

[49] Jansen A, Türck M, Szekat C, Nagel M, Clever I, Bierbaum G. 2007. Role of insertion elements and yycFG in the development of decreased susceptibility to vancomycin in Staphylococcus aureus. Int J Med Microbiol 297:205–215. doi:10.1016/j.ijmm.2007.02.002 17418637

[50] Fabret C, Hoch JA. 1998. A two-component signal transduction system essential for growth of Bacillus subtilis: implications for anti-infective therapy. J Bacteriol 180:6375–6383. doi:10.1128/JB.180.23.6375-6383.1998 9829949 PMC107725

[51] Cameron DR, Jiang JH, Kostoulias X, Foxwell DJ, Peleg AY. 2016. Vancomycin susceptibility in methicillin-resistant Staphylococcus aureus is mediated by YycHI activation of the WalRK essential two-component regulatory system. Sci Rep 6:30823. doi:10.1038/srep30823 27600558 PMC5013275

[52] Szurmant H, Nelson K, Kim E-J, Perego M, Hoch JA. 2005. YycH regulates the activity of the essential YycFG two-component system in Bacillus subtilis. J Bacteriol 187:5419–5426. doi:10.1128/JB.187.15.5419-5426.2005 16030236 PMC1196008

[53] Szurmant H, Mohan MA, Imus PM, Hoch JA. 2007. YycH and YycI interact to regulate the essential YycFG two-component system in Bacillus subtilis. J Bacteriol 189:3280–3289. doi:10.1128/JB.01936-06 17307850 PMC1855854

[54] Dobihal GS, Brunet YR, Flores-Kim J, Rudner DZ. 2019. Homeostatic control of cell wall hydrolysis by the WalRK two-component signaling pathway in Bacillus subtilis. Elife 8:e52088. doi:10.7554/eLife.52088 31808740 PMC7299342

[55] Poon R, Basuino L, Satishkumar N, Chatterjee A, Mukkayyan N, Buggeln E, Huang L, Nair V, Argudín MA, Datta SK, Chambers HF, Chatterjee SS. 2022. Loss of GdpP function in Staphylococcus aureus leads to β-lactam tolerance and enhanced evolution of β-lactam resistance. Antimicrob Agents Chemother 66:e0143121. doi:10.1128/AAC.01431-21 34843389 PMC8846394

[56] Sommer A, Fuchs S, Layer F, Schaudinn C, Weber RE, Richard H, Erdmann MB, Laue M, Schuster CF, Werner G, Strommenger B. 2021. Mutations in the gdpP gene are a clinically relevant mechanism for β-lactam resistance in meticillin-resistant Staphylococcus aureus lacking mec determinants. Microb Genom 7. doi:10.1099/mgen.0.000623 PMC8715439 34486969

[57] Dengler Haunreiter V, Tarnutzer A, Bär J, von Matt M, Hertegonne S, Andreoni F, Vulin C, Künzi L, Menzi C, Kiefer P, Christen P, Vorholt JA, Zinkernagel AS. 2023. C-di-AMP levels modulate Staphylococcus aureus cell wall thickness, response to oxidative stress, and antibiotic resistance and tolerance. Microbiol Spectr 11:e0278823. doi:10.1128/spectrum.02788-23 37948390 PMC10715141

[58] Yin W, Cai X, Ma H, Zhu L, Zhang Y, Chou SH, Galperin MY, He J. 2020. A decade of research on the second messenger c-di-AMP. FEMS Microbiol Rev 44:701–724. doi:10.1093/femsre/fuaa019 32472931 PMC7850090

[59] Fahmi T, Port GC, Cho KH. 2017. C-di-AMP: an essential molecule in the signaling pathways that regulate the viability and virulence of Gram-positive bacteria. Genes (Basel) 8:197. doi:10.3390/genes8080197 28783096 PMC5575661

[60] Pham TH, Liang ZX, Marcellin E, Turner MS. 2016. Replenishing the cyclic-di-AMP pool: regulation of diadenylate cyclase activity in bacteria. Curr Genet 62:731–738. doi:10.1007/s00294-016-0600-8 27074767

[61] Wang X, Davlieva M, Reyes J, Panesso D, Arias CA, Shamoo Y. 2017. A novel phosphodiesterase of the GdpP family modulates Cyclic di-AMP levels in response to cell membrane stress in daptomycin-resistant enterococci. Antimicrob Agents Chemother 61. doi:10.1128/AAC.01422-16 PMC5328519 28069645

[62] Commichau FM, Heidemann JL, Ficner R, Stülke J. 2019. Making and breaking of an essential poison: the cyclases and phosphodiesterases that produce and degrade the essential second messenger cyclic di-AMP in bacteria. J Bacteriol 201:e00462-18. doi:10.1128/JB.00462-18 30224435 PMC6287462

[63] Pal AK, Ghosh A. 2022. C-di-AMP signaling plays important role in determining antibiotic tolerance phenotypes of Mycobacterium smegmatis. Sci Rep 12:13127. doi:10.1038/s41598-022-17051-z 35907936 PMC9338955

[64] Kundra S, Lam LN, Kajfasz JK, Casella LG, Andersen MJ, Abranches J, Flores-Mireles AL, Lemos JA. 2021. C-di-AMP is essential for the virulence of Enterococcus faecalis. Infect Immun 89:e0036521. doi:10.1128/IAI.00365-21 34424750 PMC8519298

[65] Kussell E. 2013. Evolution in microbes. Annu Rev Biophys 42:493–514. doi:10.1146/annurev-biophys-083012-130320 23654305

[66] Luther MK, Arvanitis M, Mylonakis E, LaPlante KL. 2014. Activity of daptomycin or linezolid in combination with rifampin or gentamicin against biofilm-forming Enterococcus faecalis or E. faecium in an in vitro pharmacodynamic model using simulated endocardial vegetations and an in vivo survival assay using Galleria mellonella larvae. Antimicrob Agents Chemother 58:4612–4620. doi:10.1128/AAC.02790-13 24867993 PMC4136052

[67] Lenski RE, Travisano M. 1994. Dynamics of adaptation and diversification: a 10,000-generation experiment with bacterial populations. Proc Natl Acad Sci U S A 91:6808–6814. doi:10.1073/pnas.91.15.6808 8041701 PMC44287

[68] CLSI. 2019. Methods for dilution antimicrobial susceptibility tests for bacteria that grow aerobically; approved standard (M07-A11). Clinical and Laboratory Standards Institute, Wayne, PA, USA.

[69] Bankevich A, Nurk S, Antipov D, Gurevich AA, Dvorkin M, Kulikov AS, Lesin VM, Nikolenko SI, Pham S, Prjibelski AD, Pyshkin AV, Sirotkin AV, Vyahhi N, Tesler G, Alekseyev MA, Pevzner PA. 2012. SPAdes: a new genome assembly algorithm and its applications to single-cell sequencing. J Comput Biol 19:455–477. doi:10.1089/cmb.2012.0021 22506599 PMC3342519

[70] Seemann T. 2014. Prokka: rapid prokaryotic genome annotation. Bioinformatics 30:2068–2069. doi:10.1093/bioinformatics/btu153 24642063

[71] Deatherage DE, Barrick JE. 2014. Identification of mutations in laboratory-evolved microbes from next-generation sequencing data using breseq. Methods Mol Biol 1151:165–188. doi:10.1007/978-1-4939-0554-6_12 24838886 PMC4239701

