# Supplementary figures and images for: Evolutionary trajectories of β-lactam resistance in Enterococcus faecalis strains
Source: mBio. 2024 Nov 14;15(12):e02897-24. doi: 10.1128/mbio.02897-24 (PMC11633384; doi:10.1128/mbio.02897-24)

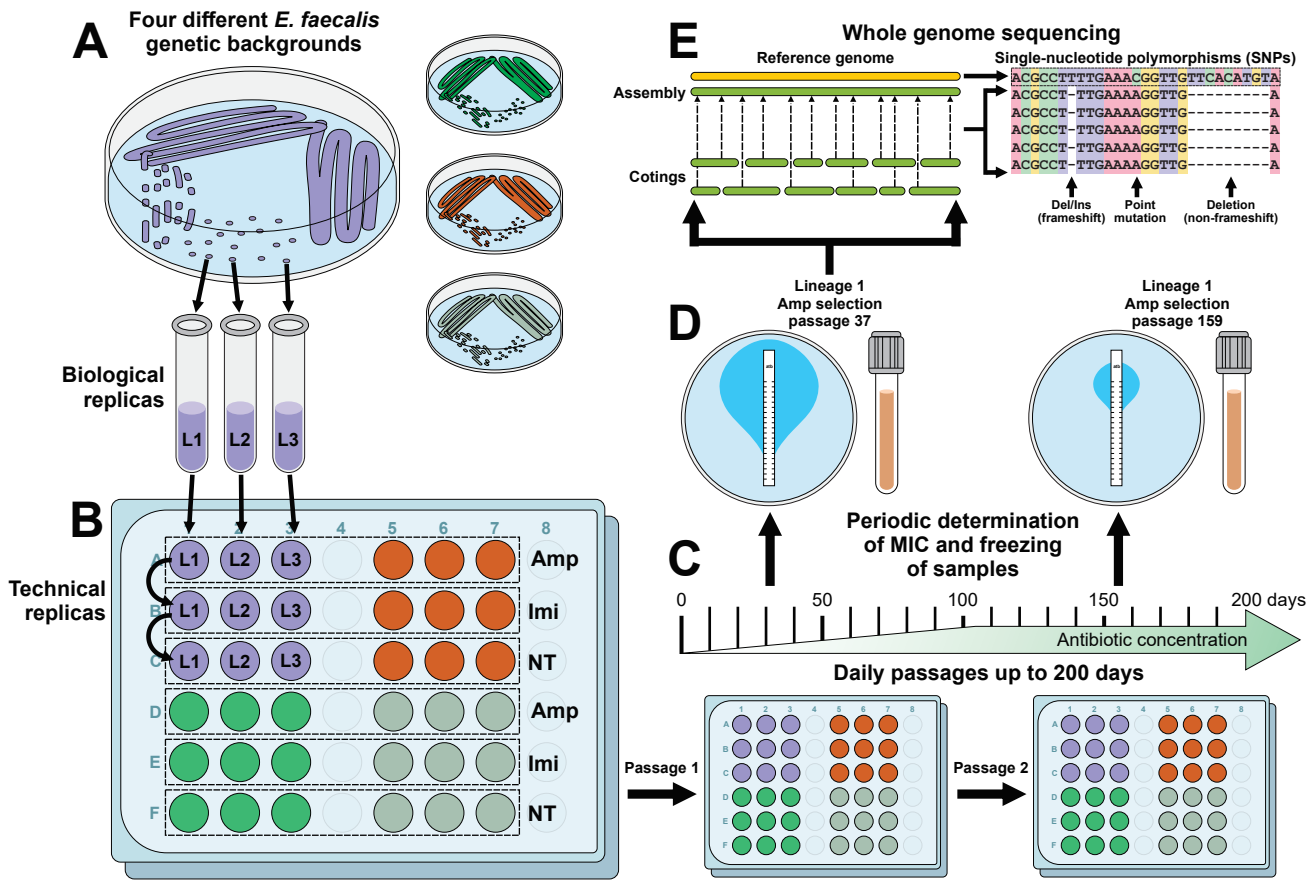

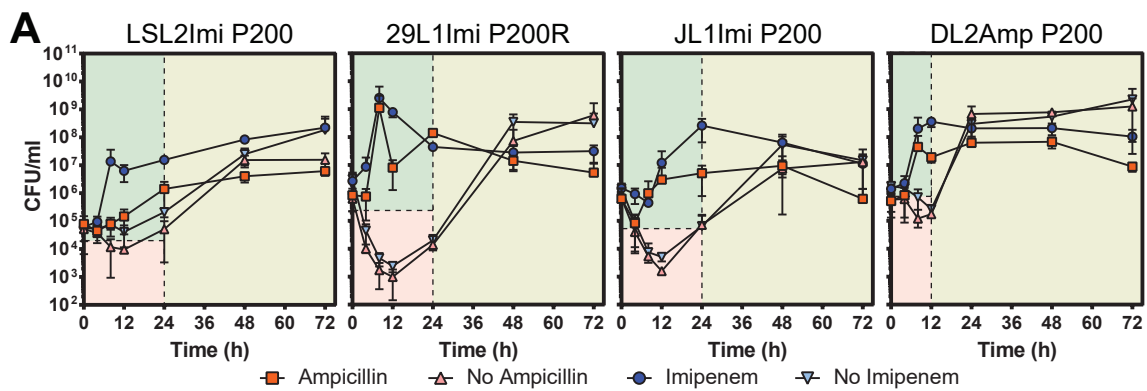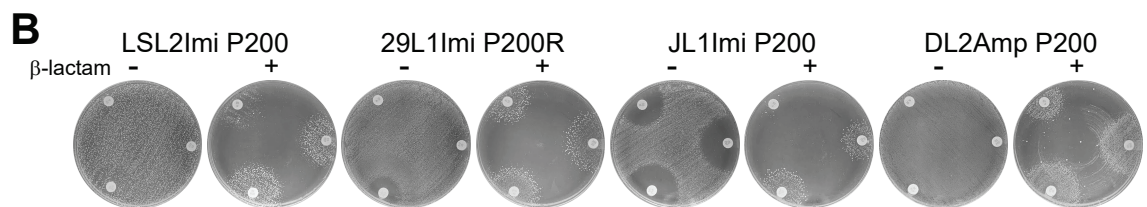

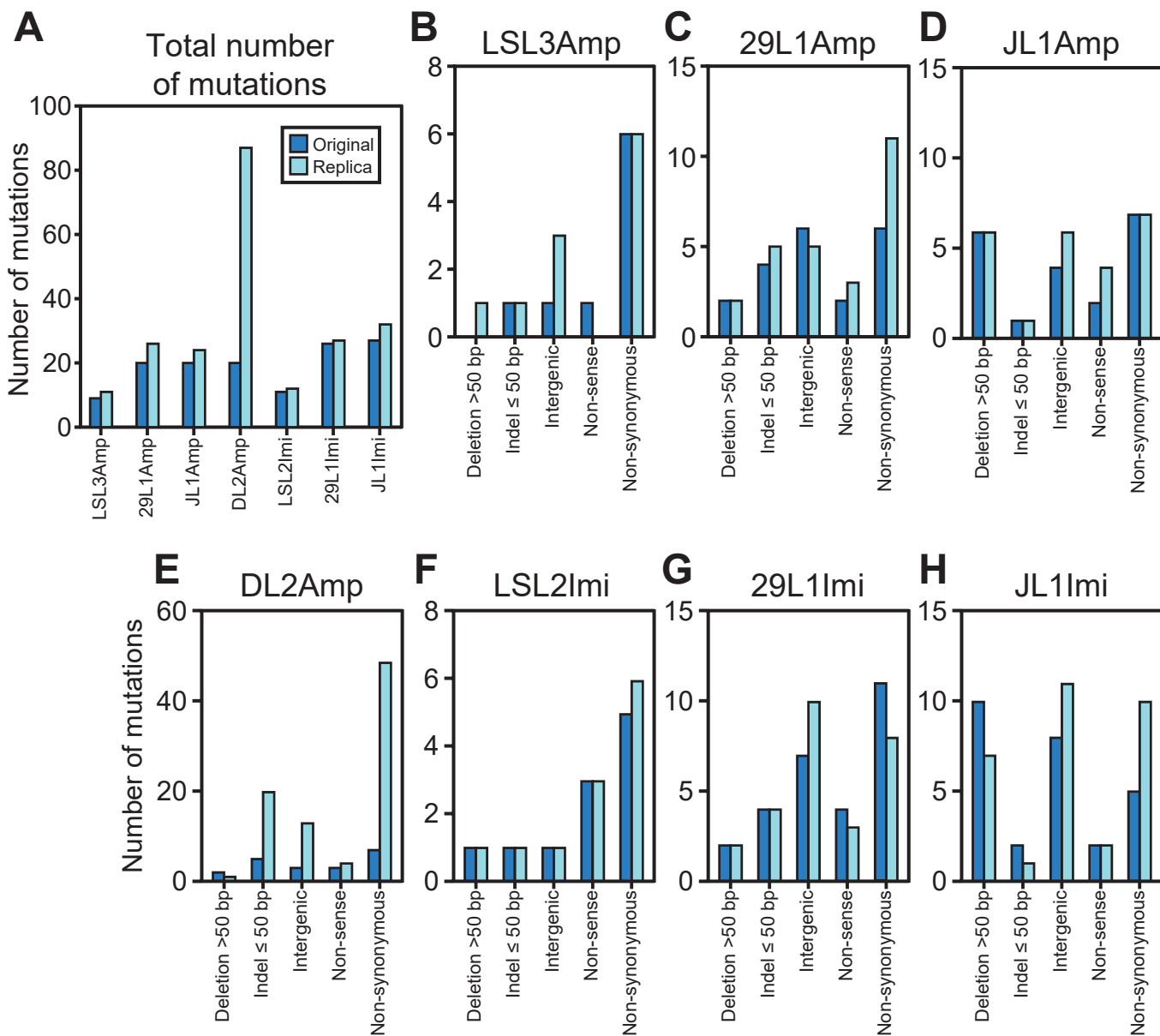

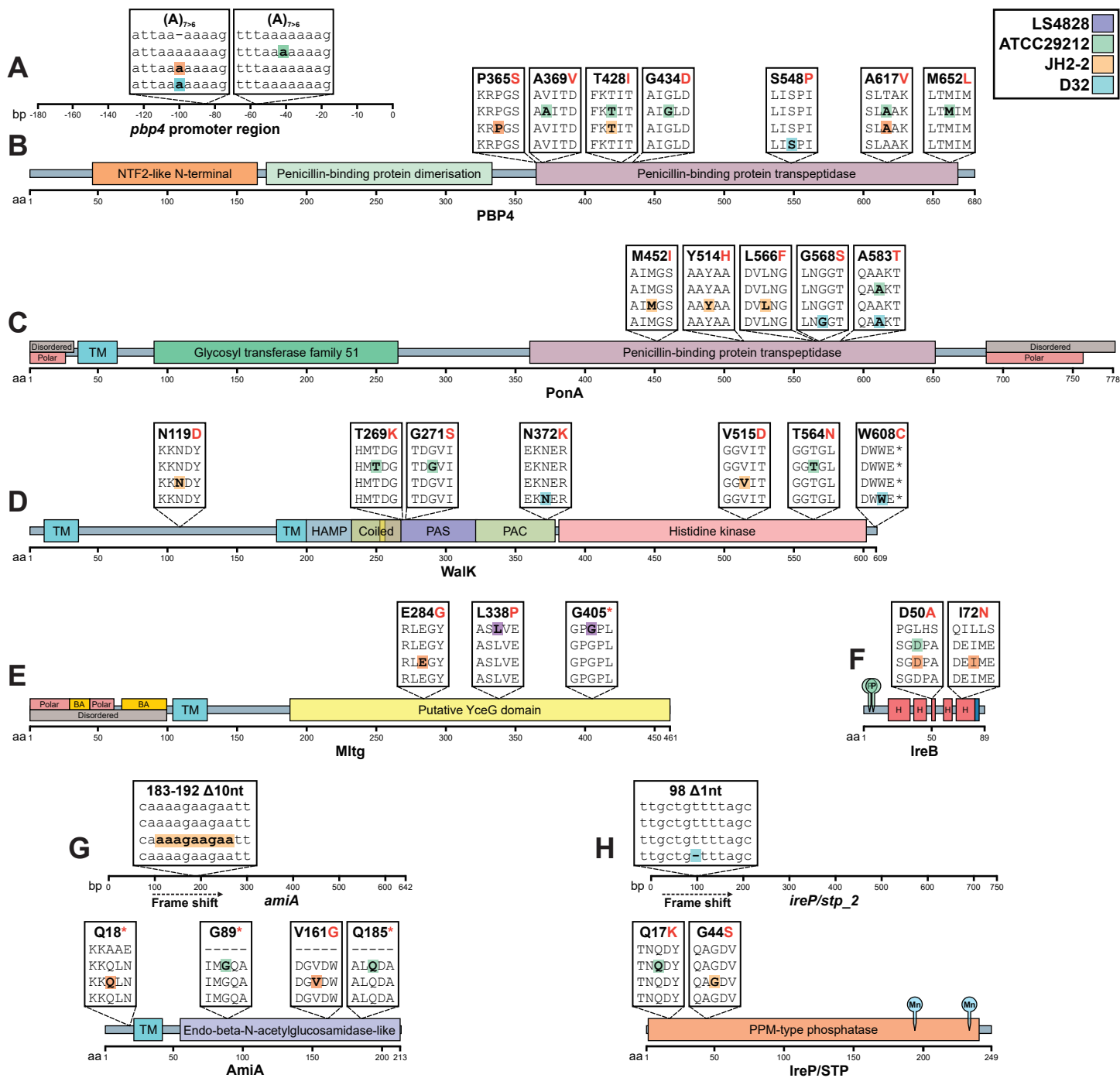

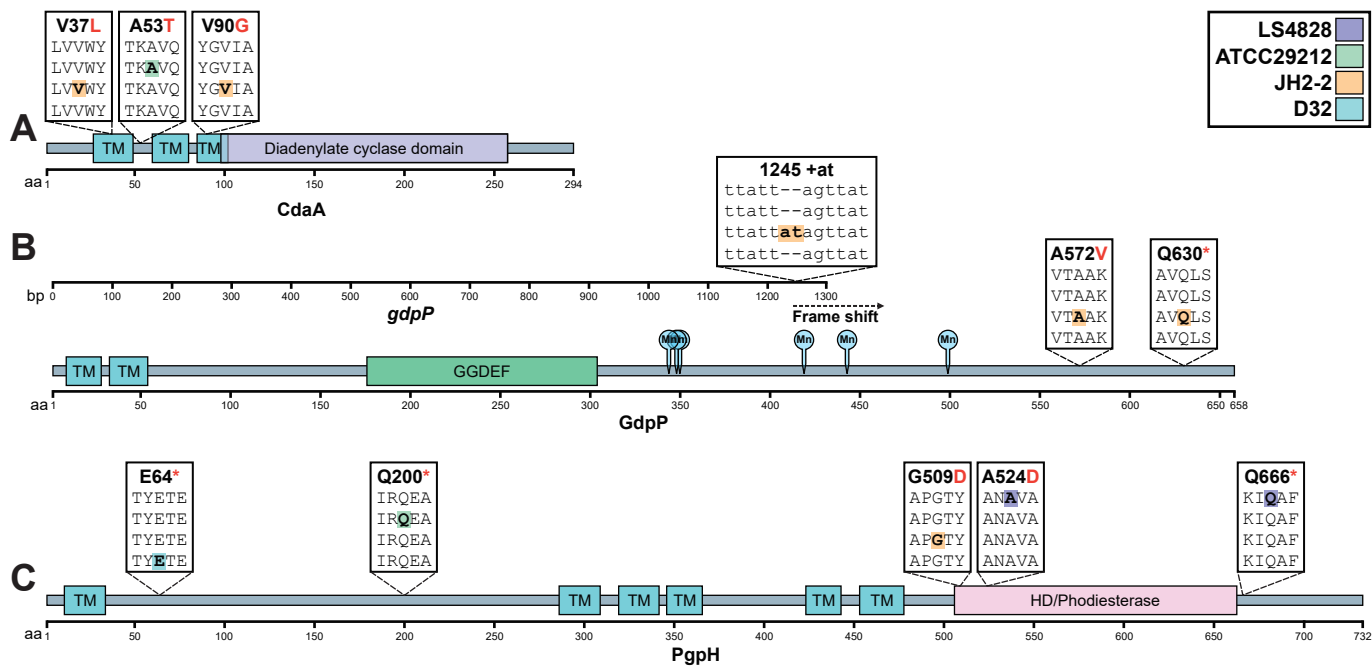

Supplement: Supplemental figures — Fig. S1 to S5. [file mbio.02897-24-s0001.pdf]
