# Supplementary material for: Evolutionary trajectories of β-lactam resistance in Enterococcus faecalis strains
Source: mBio. 2024 Nov 14;15(12):e02897-24. doi: 10.1128/mbio.02897-24 (PMC11633384; doi:10.1128/mbio.02897-24)
Supplement: Supplemental text — Supplemental methods and legends. [file mbio.02897-24-s0006.docx]

**Supplemental Material**

**Supplemental Methods**

**Bacterial Strains.** We used four different *E. faecalis* strains for which whole genome information was available and were isolated from diverse sources (Table 1): D32 (25) lacks distinct virulence-associated traits (66). D32 belongs to ST40; strains from this sequence type do not show ecological preference and have been isolated from animals, food, and humans (67). ATCC 29212 (68, 69), it belongs to ST30, which is associated with humans and dates back to at least the 1950s (67, 70) and contains several described virulence factors (69). JH2-2 (24), has been long used as a laboratory strain. LS4828 is an ampicillin resistant strain that was isolated from a prosthetic knee after prolonged treatment with amoxicillin. LS4828 overexpresses *pbp4* and the PBP4 protein has reduced affinity to β-lactams (9).

**Antimicrobials.** Ampicillin (Sigma Aldrich, St. Louis, MO), Imipenem (Combi-Blocks, San Diego, CA), MIC test strips were acquired from Liofilchem: ampicillin 0.016-256 µg/ml, ampicillin 0.002-32 µg/ml, imipenem 0.016-256 µg/ml, imipenem 0.002-32 µg/ml, penicillin 0.016-256 µg/ml, penicillin 0.002-32 µg/ml. Antibiotic discs were acquired from BD BBLTM.

**Media.** As per Clinical and Laboratory Standards Institute (CLSI) guidance, Mueller-Hinton II broth (MHII; Becton Dickinson, Sparks, MD, USA), adjusted to 25 mg/L calcium and 12.5 mg/L magnesium, was used for all susceptibility testing as previously described (71-74). Liquid culture for the LTEE and streaks were grown with brain heart infusion (BHI) (Sigma Aldrich, St. Louis, MO) due to better growth (9, 75, 76). For colony counts, MHII agar plates were used for better resolution to count.

**Minimal inhibitory concentration (MIC) testing.** 10 µl from each sample was diluted in 3 ml of BHI broth. The entire surface of regular BHI agar Petri plate was inoculated in three different directions by using cotton swabs dipped in the inoculum suspensions. MIC strip tests were applied onto the inoculated agar using forceps. The plates were incubated at 37° C and read by eye after 18-24 h of incubation.

**Disc diffusion assays.** Overnight cultures were adjusted to OD_600_ 0.01 of in 3 ml of BHI broth supplemented with either ampicillin or imipenem, depending on the antibiotic selection and concentrations used during the LTEE. The cultures were incubated until they reached OD_600_ an ~0.5. The OD_600_ was adjusted to 0.01. The entire surface of regular BHI agar Petri plate was inoculated in three different directions by using cotton swabs dipped in the inoculum suspensions. Disks were applied to the inoculated agar by using forceps. The plates were incubated at 37° C and the inhibition zone was measured after 24, 48,72 and 96 h.

**Growth Curves.** Lineages were streaked fresh from -80º C stored glycerol stocks onto BHI agar Petri plates. An ampicillin or imipenem disc was placed on the center of the agar plate. Additionally, complemented lineages were inoculated onto BHI agar Petri plates supplemented with spectinomycin at 300 µg/ml. The plates were incubated for 18 h at 37° C (or longer in the case of slow-growing dependent lineages). Three to five colonies were picked up near the disc and inoculated into BHI broth supplemented with either ampicillin or imipenem. Cultures were incubated at 37° C overnight or longer depending on growth rate. Fresh 20 ml of BHI supplemented with the corresponding β-lactam were inoculated at an OD_600_ 0.01 and incubated in a shaking incubator at 37° C and 180 rpm. Growth was monitored by OD_600_ at 0, 4, 8, 12, 24, 36, and 48 h.

For CFU counts, overnight cultures were washed three times with saline solution to clean remains of antibiotic used for growth overnight. Cultures were inoculated at an initial OD_600_ 0.01 into 5 ml of fresh BHI broth supplemented with or without the corresponding β-lactam. The cultures were incubated in a shaking incubator at 37° C and 180 rpm. Samples were obtained at 0, 4, 8, 12, 24, 36, and 48 h. Samples were serially diluted and plated onto MHI agar plates to count and calculate the CFU per ml. The plates were supplemented with 5 µg/ml of ampicillin for β-lactam dependent lineages. The lower limit of detection (LLD) was 2.0 log10 CFU/ml.

**Plasmid construction and transformation.**

The coding region of LS4828 *pgpH* was cloned by PCR and restriction digestion into the BamHI and XbaI sites of pBSU101 (77), producing a fusion of the pgpH CDS with the plasmid CFB promoter. The sequences of the plasmid were confirmed, and they were transformed into *E. faecalis* by electroporation.

Electrocompetent cells were prepared according to the protocol of Friesenegger et al. 1991 (78) with modifications. Cells were grown in Todd-Hewitt broth supplemented with the corresponding antibiotic used during the LTEE. Overnight cultures were diluted 1:1000 in 150 mL of Todd-Hewitt broth and incubated for 12 hours or until stationary phase was reached. Cells were harvested by centrifugation at 3800 x g for 15 minutes and washed in cold 10% glycerol with 1/1, ½, ¼ and 1/8 of the initial culture volume. The cell pellets were resuspended in 1 mL of 10% glycerol, aliquoted, and frozen at -80^o^C. Electroporation was achieved by following the protocol of (78). Fifty microliter aliquots of electrocompetent cells were thawed on ice and mixed with ~500 ng of pBSU101 or *p*pgpH DNA. Mixes were transferred to an ice-cooled 0.2 cm cuvette and pulsed with field strength of 12.500 V/cm using the 400Ω resistor to result in time constants of 9-16 ms. Cells were recovered in 1 mL Todd-Hewitt broth for 1 hr. at 37^o^C with 180 rpm shaking. One-hundred or 300 microliters of cells were spread onto BHI agar plates supplemented with 300 µg/mL spectinomycin and 5 µg/mL ampicillin. The plates were incubated for 48 hr. at 37^o^C to allow growth of transformants for selection. Colonies were passed one time to a fresh BHI agar plate plus spectinomycin 150 ug/mL and an ampicillin disc (10 µg) placed centrally prior to growing the transformants in broth for glycerol stocks and experiments.

**Genomic extraction.** Genomic DNA was extracted from 2 mL of culture at mid log phase (OD_600_ of 0.500) using the Monarch Genomic Minikit (New England Biolabs, MA), following the manufacturer’s protocol with the following modifications: cells were lysed with 10 mg/mL lysozyme in Tris-EDTA pH 8 for 1 hour at 37ºC. The DNA library was prepared using the TruSeq DNA PCR free kit 350 (Illumina) following the manufacturer's user guide. The initial concentration of DNA was evaluated using the Qubit® dsDNA HS Assay Kit (Life Technologies), and 50 ng DNA was used to prepare the library.

**Preparation of samples for c-di-AMP Competitive ELISA.** Parental strains and evolved lineages were grown and prepared for intracellular cyclic di-AMP measurements with some modifications according to the protocol of Wang et al. 2017 (61). Parental strains and evolved lineages were inoculated from glycerol stocks into 3 ml of regular BHI broth or BHI supplemented with ampicillin or imipenem, depending on the antibiotic or concentration used during the LTEE, and incubated with shaking at 180 rpm at 37° C for 17-48 h, depending on the strain, passage, or lineage. After incubation, cultures were diluted back 1:50 or 1:100 into 55 mL BHI supplemented with the corresponding antibiotic and grown with shaking at 180 rpm at 37° C to late exponential phase (OD_600_ ~1.0). A minimum of 3 independent biological replicates were obtained for analysis.

Cell pellets from 45 ml culture volumes were centrifuged at 4000 g for 20 min and washed 3 times with 5 ml phosphate-buffered saline (PBS) followed by centrifugation at 4000 g for 5 min., pouring off PBS and freezing the cell pellet at -20oC. The cells were re-suspended in 0.5 ml of 50 mM Tris-HCL pH 8.0 and broken using Lysing Matrix B tubes (Thermo Fisher Scientific, Waltham, MA) with a BioSpec Mini Beadbeater-16 bead for 20 sec five times, with 1 minute on ice in-between. Cell lysates were collected by centrifuging for 15 min at 12,000 g. The lysate was transferred to a new tube, removing a small sample for protein concentration (Pierce BCA protein assay kit; Thermo Fisher Scientific). Protein concentrations were used to confirm complete cell lysis of samples. The remaining supernatant was boiled at 95° C for 5 min, cooled, and collected by centrifugation at 12,000 g for 15 min at 4° C. The protein-free supernatants were stored at -80° C until run in the competitive ELISA specific for c-di-AMP.

**Supplemental Tables**

**Supplemental Table S1. Lineage identification key and sequenced genomes.**

**Supplemental Table S2. Ampicillin and imipenem MICs from lineages with no selective pressure.** Heat map of the Minimal Inhibitory Concentration (MIC) of ampicillin (red) and imipenem (blue) obtained from the lineages evolved without antibiotic selective pressure. MICs were determined between 18-24h of incubation and read by eye. The vertical dotted line delimits the passages from the original culture and the passages from the replica culture.

**Supplemental Table S3. Disc diffusion assays.** Disc diffusion assay in dependent lineages at passage 200 to determine resistance/susceptibility and dependence. β-lactam dependency is observed with different groups of β-lactams. Disc diffusion assays with penicillin (P-10 µg), oxacillin (OX-1 µg), ticarcillin (TIC-75 µg), piperacillin (PRL-100 µg), ampicillin (AM-10 µg), imipenem (IPM-10 µg), cefazolin (KZ-30 µg), cefuroxime (CXM-30 µg), cefotetan (CTT-30 µg), ceftazidime (CAZ-30 µg), ceftriaxone (CRO-30 µg), cefaclor (CEC-30 µg), cefepime (FEP-30 µg), and vancomycin (VA-30 µg). Mean diameter for three independent experiments. Cells were plated onto BHI agar Petri dishes at an OD600 of 0.01. The diameter of the growth or inhibitory halos were measured at 24, 48, 72, and 96 h.

**Supplemental Table S4. Mutations identified in genes related to cell wall synthesis and degradation.**

**Supplemental Table S5. Mutations in genes of the c-di-AMP biosynthetic pathway.**

**Supplemental Table S6. Protein accession numbers.**

**Supplemental datasets.** List of mutations identified in the lineages generated in the LTEE at different timepoints.

**Supplemental Figures**

**Supplemental Figure 1. Set up of the long-term evolutionary experiment (LTEE). A)** Three single colonies from each strain were picked up from BHI agar plates and seeded into three independent cultures (5 ml of BHI broth) establishing three lineages per strain (L1, L2, and L3) as biological replicas. **B)** Each founding lineage was split into three conditions: ampicillin (Amp), imipenem (Imi), or no treatment (NT). **C)** Plates were incubated at 37°C with shaking at 180 rpm for 24 h. Serial passages were performed daily by transferring 5 µl of each well into 500 µl of fresh BHI with the corresponding antibiotic concentrations. The experiment consisted of 200 consecutive daily passages. Amp or Imi concentrations were increased gradually up to day 104, and then it remained constant until day 200. **D)** Periodic freezing of samples and determination of MICs were performed. Amp and Imi MICs were determined by MIC strip test. **E)** Genomic DNA was obtained from specific samples to perform whole genome sequencing and variant calling.

**Supplemental figure 2. β-lactam dependent lineages grow dynamics.** The dependent lines grew faster in the presence of β-lactams. **A)** Growth curves with CFU/ml count of four β-lactam dependent lineages from passage 200 (P200): LSL2Imi P200OR (LS4828 background, original culture), 29L1Imi P200NRP (ATCC29212, replica culture), JL1Imi P200OR (JH2-2 background, original culture), and DL2Amp P200 (D32 background, original culture). The cells were grown overnight (ON) in the presence of ampicillin or imipenem. The ON cultures were washed three times with saline solution and adjusted to an initial 0.01 OD_600_ in BHI broth. The adjusted cultures were grown in the presence or absence of either ampicillin or imipenem using 118 ug/ml for LSL2imi, and 29 ug/ml for the other backgrounds, the same concentrations were used during the LTEE for those specific lineages. Samples were taken at 0, 4, 8, 12, 24, 48, and 72 h for CFU counts. The upper left area of the graph highlights the exponential growth phase in the presence of β-lactams. The lower left shows the population decline in the absence of antibiotic. The right side of the graph highlights the lag in exponential growth phase of the cultures without antibiotic and the stationary phase. **B)** Disc diffusion assay with ampicillin (Right side of the plate, AM-10 µg), imipenem (Bottom left of the plate, IPM-10 µg), and penicillin (Top left of the plate, P-10 µg). Cells were grown in BHI broth with or without the antibiotic in which they were originally selected until an 0.5 OD_600_. The cells were diluted to an 0.01 OD_600_ and streaked onto BHI agar Petri dishes. LSL2Imi P200 and DL2Amp P200 grew throughout the plate without a clearing inhibition zone. Lineage 29L1Imi P200R had inhibitory halos of 1.3 ± 0.57 mm to ampicillin, 4 ± 1 mmm to imipenem, and penicillin resistance, lineage JL1Imi P200 had inhibitory halos of 10.3 ± 0.57 mm to ampicillin, 9.6 ± 1.5 mmm to imipenem, and 7.3 ± 1.1 mm to penicillin. An inhibition zone diameter ≥ 15 mm indicates penicillin susceptibility and ≥ 17 mm indicates susceptibility to other β-lactams.

**Supplemental figure S3. Quantity and type of mutations detected in sequenced evolved lineages.** Summary of the number and type of mutations accumulated over time by seven selected lineages. **A)** Total number of mutations acquired by the lineages in the original (dark blue) and replica (light blue) culture. The mutations were grouped into five categories depending on the genetic change: deletions >50 bp, indel ≤50 bp, intergenic, non-sense, and non-synonymous. The number of mutations per category was counted in the genomes of **A)** LSL3Amp, **C)** 29L1Amp, **D)** JL1Amp, **E)** DL2Amp, **F)** LSL2Imi, **G)** 29L1Imi, and **H)** JL1Imi.

**Supplemental figure S4. Mutations in genes related to β-lactam resistance and cell wall regulation identified in more than one resistant lineage.** **A)** *pbp4* promoter region with the detected mutations, positions are relative to the gene start codon. **B)** Pbp4 domain organization and mutated residues. **C)** PonA mutations. **D)** WalK mutations. **E)** Mltg mutations. **F)** IreB mutations, LS4828-*ireB* has an insertion of an A at position 51 of the CDS, causing a frameshift in the protein. **G)** IreP mutations. Squares express the alignment of the mutated regions from the four different genetic backgrounds. The corresponding mutations are highlighted in red. Asterisks srepresent premature STOP codons. The mutated base or amino acids are bolded and squared within the alignment. Purple: LS4828, green: ATCC29212, orange: JH2-2, and blue: D32.

**Supplemental figure 5. Mutations identified in the c-di-AMP** **synthesis and degradation genes.** Domain organization (aa) of the **A)** adenylate cyclase CdaA, and the phosphodiesterases **B)** GdpP and **C)** PgpH. Squares express the alignment of the mutated regions from the four different genetic backgrounds. The corresponding mutations are highlighted in red asterisks represent premature STOP codons. The mutated amino acids are bolded and highlighted within the alignment. Putative binding regions, TM domains and active domains are highlighted for each protein. Purple: LS4828, green: ATCC29212, orange: JH2-2, and blue: D32. Line on the top of GdpP protein (B) represents the genetic position (bp) of the gene with a mutation that caused a frameshift.
